# Functional characterization of phospholipase C-γ_2_ mutant protein causing both somatic ibrutinib resistance and a germline monogenic autoinflammatory disorder

**DOI:** 10.18632/oncotarget.26173

**Published:** 2018-09-28

**Authors:** Claudia Walliser, Martin Wist, Elisabeth Hermkes, Yuan Zhou, Anja Schade, Jennifer Haas, Julia Deinzer, Laurent Désiré, Shawn S.C. Li, Stephan Stilgenbauer, Joshua D. Milner, Peter Gierschik

**Affiliations:** ^1^ Institute of Pharmacology and Toxicology, Ulm University Medical Center, Ulm 89070, Germany; ^2^ Diaxonhit, Paris 75013, France; ^3^ Department of Biochemistry and The Siebens-Drake Medical Research Institute, Schulich School of Medicine, University of Western Ontario, London, Ontario N6A 5C1, Canada; ^4^ Department of Internal Medicine III, Ulm University Medical Center, Ulm 89070, Germany; ^5^ Laboratory of Allergic Diseases, NIAID, NIH, Bethesda, MD 20892, USA

**Keywords:** chronic lymphocytic leukemia, autoinflammation, B cell signaling, Bruton’s tyrosine kinase, inositol phosphates

## Abstract

Depending on its occurrence in the germline or somatic context, a single point mutation, S707Y, of phospholipase C-γ_2_ (PLCγ_2_) gives rise to two distinct human disease states: acquired resistance of chronic lymphocytic leukemia cells (CLL) to inhibitors of Brutons´s tyrosine kinase (Btk) and dominantly inherited autoinflammation and PLCγ_2_-associated antibody deficiency and immune dysregulation, APLAID, respectively. The functional relationships of the PLCγ_2_S707Y mutation to other *PLCG2* mutations causing (i) Btk inhibitor resistance of CLL cells and (ii) the APLAID-related human disease PLCγ_2_-associated antibody deficiency and immune dysregulation, PLAID, revealing different clinical characteristics including cold-induced urticaria, respectively, are currently incompletely understood. Here, we show that PLCγ_2_S707 point mutants displayed much higher activities at 37° C than the CLL Btk inhibitor resistance mutants R665W and L845F and the two PLAID mutants, PLCγ_2_Δ19 and PLCγ_2_Δ20-22. Combinations of CLL Btk inhibitor resistance mutations synergized to enhance PLCγ_2_ activity, with distinct functional consequences for different temporal orders of the individual mutations. Enhanced activity of PLCγ_2_S707Y was not observed in a cell-free system, suggesting that PLCγ_2_ activation in intact cells is dependent on regulatory rather than mutant-enzyme-inherent influences. Unlike the two PLAID mutants, PLCγ_2_S707Y was insensitive to activation by cooling and retained marked hyperresponsiveness to activated Rac upon cooling. In contrast to the PLAID mutants, which are insensitive to activation by endogenously expressed EGF receptors, the S707Y mutation markedly enhanced the stimulatory effect of EGF, explaining some of the pathophysiological discrepancies between immune cells of PLAID and APLAID patients in response to receptor-tyrosine-kinase activation.

## INTRODUCTION

Recent evidence suggests that *PLCG2* mutations are involved in several human pathologies. Deletions of exons 19 or 20–22 cause cold urticaria and PLCγ_2_–associated antibody deficiency and immune dysregulation, PLAID [1, 2], while a point mutation (S707Y) is the basis of autoinflammation and PLCγ_2_-associated antibody deficiency and immune dysregulation, APLAID [3]. In addition, several point mutations as well as small deletions have been found to mediate resistance of chronic lymphocytic leukemia (CLL) cells to the Btk inhibitor ibrutinib [4–9]. *PLCG2* point mutations have also been identified in association with childhood-onset steroid-sensitive nephrotic syndrome [10], Burkitt lymphoma [11], and inflammatory bowel disease [12]. The mutation P522R was found to be protective against late-onset Alzheimer's disease [13]. *PLCG2* mutations in position 707 are particularly intriguing, because they give rise to clinically disparate conditions: APLAID, when germline, and ibrutinib-resistant CLL, when somatic. Overexpression of the S707Y mutant in model cells have previously been shown to result in enhanced basal and EGF-receptor-stimulated inositol phosphate formation as well as increases in [Ca^2+^]_i_. *Ex vivo* experiments using affected individuals’ leukocytes showed enhanced inositol phosphate formation and increases in [Ca^2+^]_i_ upon crosslinking stimulation of cells with IgE and enhanced ERK phosphorylation following BCR ligation with anti-IgM [3]. Subsequent studies on peripheral blood mononuclear cells (PBMCs) of APLAID patients suggested that the S707Y mutation of *PLCG2* contributes to the activation of the NOD-like receptor (NLR) family, pyrin domain–containing protein 3 (NLRP3) inflammasome in these patients, presumably by promoting, through increased [Ca^2+^]_i_, inflammasome component assembly and spontaneous inflammasome activity [14, 15].

We have previously shown that the two PLAID PLCγ_2_ mutants, PLCγ_2_Δ19 and PLCγ_2_Δ20-22, are strongly (>100-fold), rapidly, and reversibly activated by cooling to temperatures only a few degrees below 37° C. We found that the mechanism(s) underlying PLCγ_2_ PLAID mutant activation by cool temperatures is distinct from a mere loss of SH-region-mediated autoinhibition and is dependent on both the integrity and the pliability of the spPH domain [16]. Subsequently, we showed that the first two PLCγ_2_ point mutants to be described to mediate ibrutinib resistance in CLL, R665W and L845F, are strikingly hypersensitive to activation by Rac [17]. The results suggested that the *PLCG2* mutations cause ibrutinib resistance by rerouting of transmembrane signals emanating from cell surface receptors of neoplastic B cells and converging on PLCγ_2_ through Rac. Very little is known about the functional consequences of S707Y *PLCG2* mutations, their relationship to other *PLCG2* mutations causing ibrutinib resistance in hematologic malignancies or to PLAID mutations.

## RESULTS

### The identity of the substitution at residue S707 determines the degree of enhanced basal PLCγ_2_ activity in intact cells

**Figure 1:**
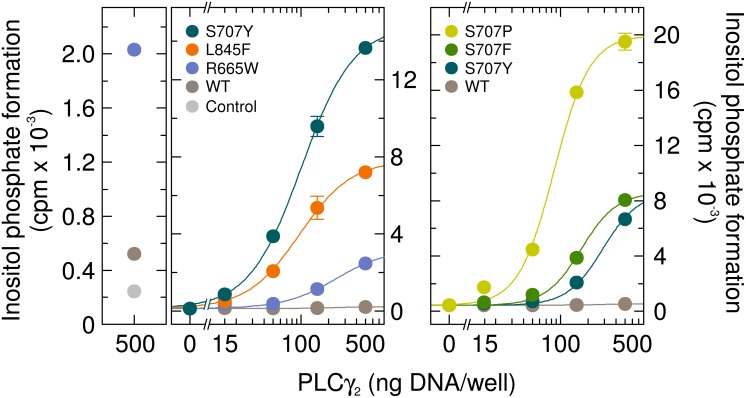
The PLCγ_2_ S707 point mutants identified in ibrutinib resistance of CLL display enhanced basal activity in cultured mammalian cells Left panels, comparison of PLCγ_2_S707Y to the PLCγ_2_ ibrutinib resistance mutants R665W and L845F. COS-7 cells were transfected as indicated with 500 ng/well of either empty vector (*Control*) or increasing amounts (15, 50, 150, and 500 ng DNA/well) of vector encoding either wild-type PLCγ_2_ (*WT*), PLCγ_2_R665W (*R665W*), PLCγ_2_L845F (*L845F*), or PLCγ_2_S707Y (*S707Y*). The results shown in the two panels are from separate experiments. Right panel, comparison to the PLCγ_2_ ibrutinib resistance mutants S707F and S707P. COS-7 cells were transfected as in the center panel, except that vectors encoding PLCγ_2_S707F (*S707F*) or PLCγ_2_S707P (*S707P*) were used where indicated at the abscissa. Twenty four hours after transfection, the cells were incubated for 18 h with *myo*-[2-^3^H]inositol, and inositol phosphate formation was then determined.

The first experiment was designed to determine the basal activity of the PLCγ_2_ mutant S707Y in intact cells and to compare this activity to two PLCγ_2_ mutants mediating resistance to ibrutinib in CLL characterized before [17], PLCγ_2_R665W and PLCγ_2_L845F (Figure 1, left panel). The three mutants were expressed in COS-7 cells and the cells were radiolabeled with [^3^H]inositol for measurement of [^3^H]inositol phosphate formation. Expression of wild-type PLCγ_2_, analyzed for comparison, had a very limited, ~ 2.1-fold stimulatory effect on basal inositol phosphate formation (Figure 1, very left). While the PLCγ_2_R665W and PLCγ_2_L845F displayed up to ~20-fold and ~61-fold enhanced basal inositol phosphate formation, respectively, the ability of PLCγ_2_S707Y to enhance basal activity was even higher, ~120-fold in this experiment (Figure 1, left panel). Two other point mutants in position 707 have been reported to occur in ibrutinib-resistant CLL patients, PLCγ_2_S707F and PLCγ_2_S707P [5]. Figure 1, right panel, shows that all three S707 mutants displayed enhanced basal enzyme activity in intact cells. While PLCγ_2_S707Y and PLCγ_2_S707F caused roughly similar maximal enhancements (~16-fold *vs.* ~19-fold, respectively), this activity was even higher (~48-fold) for PLCγ_2_S707P. Supplementary Figure 1 shows that there were only minor, if any differences in protein expression between the PLCγ_2_ variants tested in Figure 1.

### PLCγ_2_ S707 mutants are hypersensitive towards stimulation by Rac2

**Figure 2:**
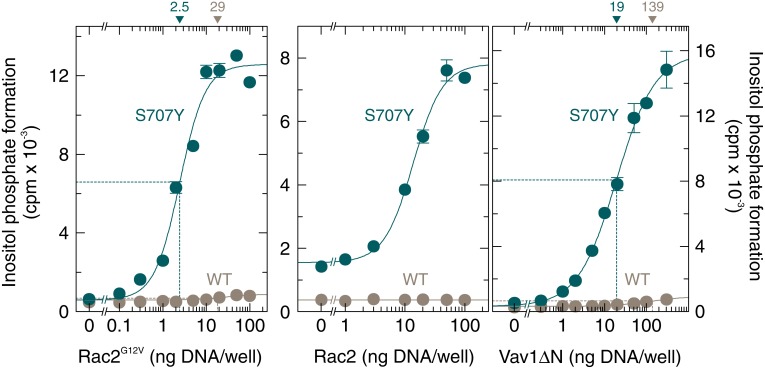
The point mutation S707Y augments the responsiveness of PLCγ_2_ to constitutively active Rac2, wild-type Rac2, and constitutively active Vav1 COS-7 cells were transfected as indicated with 50 ng/well of vector encoding wild-type PLCγ_2_ (*WT*) or PLCγ_2_S707Y (*S707Y*) and increasing amounts of vector encoding Rac2^G12V^ (left panel). Center panel, COS-7 cells were transfected as in the left panel except that 150 ng/well of vector encoding PLCγ_2_ and increasing amounts of vector encoding wild-type Rac2 was used as indicated at the abscissa. Right panel, COS-7 cells were transfected as in the left panel except that increasing amounts of vector encoding Vav1ΔN was used as indicated at the abscissa. Twenty four hours after transfection, the cells were incubated for 18 h with *myo*-[2-^3^H]inositol, and inositol phosphate formation was then determined. The ED_50_ values of vectors encoding Rac2^G12V^, Rac2, or Vav1ΔN for the stimulation of wild-type or mutant PLCγ_2_ activity obtained by non-linear curve fitting are shown *above the graphs* in nanograms/well.

To determine and compare the sensitivities of wild-type PLCγ_2_ and PLCγ_2_S707Y to regulation by Rac2, the PLCγ_2_ isozymes were co-expressed with increasing amounts of constitutively active Rac2^G12V^ (Figure 2, left), wild-type Rac2 (Figure 2, center), and constitutively active Vav1, Vav1ΔN (Figure 2, right). The latter protein presumably functions as an activator of Rac GTPases endogenously present in COS-7 cells [18]. There were striking increases in inositol phosphate formation by PLCγ_2_S707Y in response to increasing amounts of Rac2^G12V^, Rac2, and Vav1ΔN. In all three cases, the maximum increases exceeded the corresponding responses observed for wild-type PLCγ_2_ by wide margins [~21-fold, ~5.1-fold, and ~52-fold (S707Y) *vs.* ~1.5-fold, no change, and ~3.0-fold (wild-type) stimulation by Rac2^G12V^, Rac2, and Vav1ΔN, respectively]. In addition, we consistently observed that the S707Y mutation caused an increase in the apparent potency of Rac2^G12V^ and Vav1ΔN, which was ~12-fold and ~5.2-fold, respectively, in the experiment shown in Figure 2. The increase in Rac2-stimulated inositol phosphate formation caused by the S707Y mutation was not caused by changes in PLCγ_2_ protein production in transfected cells (Supplementary Figure 2A and 2B). However, we observed an about 2-fold increase in expression of exogenous wild-type and G12V mutant Rac2 upon co-expression with overactive PLCγ_2_ mutants, which may account, at least in part, for the enhanced apparent potency of Rac2 in Figure 2, left and center panels.

**Figure 3:**
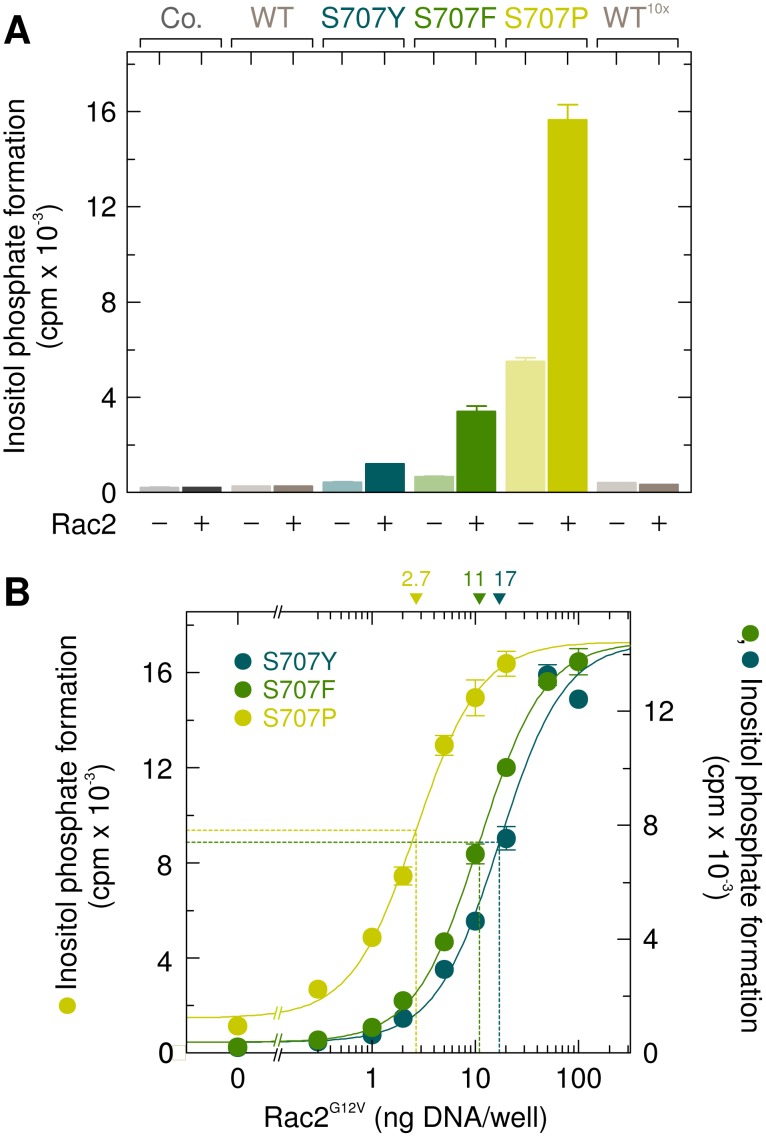
The point mutations S707Y, S707F, and S707P augment the responsiveness of PLCγ_2_ to exogenous wild-type Rac2 and Rac2^G12V^ (**A**) COS-7 cells were transfected as indicated with 50 ng or 500 ng/well (*10x*) of vector encoding wild-type PLCγ_2_ (*WT*), 50 ng/well of vector encoding PLCγ_2_S707Y (*S707Y*), PLCγ_2_S707F (*S707F*), or PLCγ_2_S707P (*S707P*) and 25 ng/well of vector encoding wild-type Rac2. Twenty four hours after transfection, the cells were incubated for 18 h with *myo*-[2-^3^H]inositol, and inositol phosphate formation was then determined. (**B**) COS-7 cells were transfected with 15 ng/well of vector encoding PLCγ_2_S707Y (*S707Y*), PLCγ_2_S707F (*S707F*), or PLCγ_2_S707P (*S707P*) and increasing amounts of vector encoding Rac2^G12V^. The ED_50_ values of vector encoding Rac2^G12V^ for the stimulation of mutant PLCγ_2_ activity obtained by non-linear curve fitting are shown *above the graphs* in nanograms/well.

A comparison of the sensitivities of the mutants PLCγ_2_S707Y, PLCγ_2_S707F, and PLCγ_2_S707P to wild-type Rac2 is shown in Figure 3A. Wild-type PLCγ_2_ was analyzed for comparison using the same (*WT*) or a 10-fold enhanced (*WT ^10x^*) amount of PLCγ_2_-encoding cDNA for transfection. While there was no response of the wild-type enzyme to wild-type Rac2 even in the presence of PLCγ_2_ at a markedly higher amount (*WT ^10x^*), inositol phosphate formation was enhanced by Rac2 in cells expressing mutants PLCγ_2_S707Y, PLCγ_2_S707F, and PLCγ_2_S707P by about ~2.7-fold, ~5.0-fold, and ~2.8-fold, respectively. The expression of mutant PLCγ_2_ enzymes was similar to that of wild-type PLCγ_2_, regardless of the presence of exogenous Rac2 in the cells (Supplementary Figure 3). In the next experiment, PLCγ_2_S707Y, PLCγ_2_S707F, and PLCγ_2_S707P were expressed at a lower density to avoid consumption of the available phospholipid substrate at high phospholipase C activities and reconstituted with increasing concentrations of Rac2^G12V^. Figure 3B shows that Rac2^G12V^ caused a marked, concentration-dependent enhancement of inositol phosphate formation by all three PLCγ_2_ mutants. Due to the higher basal activity of PLCγ_2_S707P, this mutant revealed a lower fold-stimulation by Rac2^G12V^ than the mutants PLCγ_2_S707F and PLCγ_2_S707Y (11.7-fold *vs.* 38.2-fold). Interestingly, PLCγ_2_S707P was 4.0-fold and 6.3-fold more sensitive to Rac2^G12V^ than PLCγ_2_S707F and PLCγ_2_S707Y, respectively, as shown by decreases in the amounts of Rac2^G12V^-encoding cDNA required to achieve half-maximal activation from 17 ng/well, to 11 ng/well, and 2.7 ng/well.

### The enhanced basal activity of PLCγ_2_S707Y is dependent on the activity of endogenously expressed Rac

**Figure 4:**
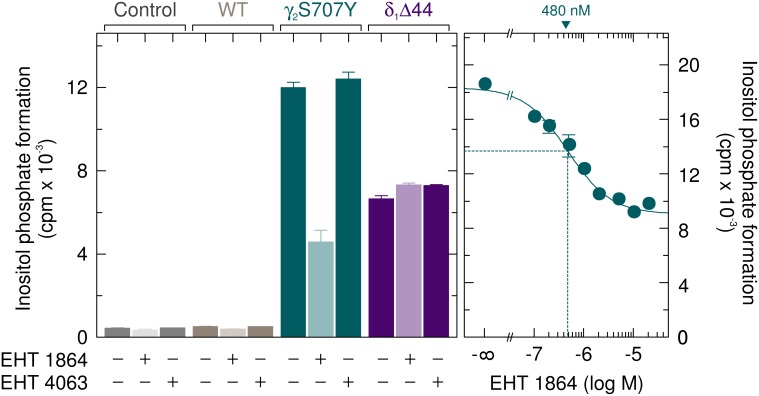
The enhanced basal activity of PLCγ_2_S707Y is specifically reduced by the Rac inhibitor EHT 1864 COS-7 cells were transfected with 500 ng/well of empty vector (*Control*) or vector encoding wild-type PLCγ_2_ (*WT*) or PLCγ_2_S707Y (*S707Y*), or 100 ng/well of vector encoding PLCδ_1_Δ44 (δ_1_*Δ44*). Twenty four hours after transfection, the cells were incubated for 18 h with *myo*-[2-^3^H]inositol in the absence or presence of 5 μM EHT 1864 or 5 μM of its inactive congener EHT 4063, followed by determination of inositol phosphate formation (left panel). In the right panel, COS-7 cells were transfected with 500 ng/well of vector encoding PLCγ_2_S707Y. Twenty four hours after transfection, the cells were incubated for 18 h with *myo*-[2-^3^H]inositol in the absence or presence of EHT 1864 at the concentrations indicated at the *abscissa*. The IC_50_ value of EHT 1864 for the inhibition of mutant PLCγ_2_ activity obtained by non-linear curve fitting is shown *above the graph* in nanomolar.

We have previously used the Rac inhibitor EHT 1864 and its inactive analog EHT 4063 to investigate the involvement of activated endogenously expressed Rac in enhanced basal activity of the PLCγ_2_ ibrutinib-resistance mutants R665W and L845F [17]. Figure 4, left panel, shows that EHT 1864, but not EHT 4063, caused a clear (~62 %) inhibition of basal inositol phosphate formation by PLCγ_2_S707Y. There was a smaller (~24 %), statistically significant (*p* = 0.0067) inhibitory effect for wild-type PLCγ_2_. No inhibitory effect of EHT 1864 was observed in the absence of exogenous PLC isozyme and in the presence of PLCδ_1_Δ44, a constitutively active variant of Rac-insensitive PLCδ_1_. The inhibitory effect of EHT 1864 on basal inositol phosphate formation by PLCγ_2_S707Y was concentration-dependent with an IC_50_ of about 0.5 μM (Figure 4, right panel), which is slightly lower than the value reported previously for PLCγ_2_L845F of about 1 μM [17]. There was little, if any effect of EHT 1864 and EHT 4063 on the expression of the various recombinant PLC isozymes and of Rac1 endogenously present in transfected cells (Supplementary Figure 4).

**Figure 5:**
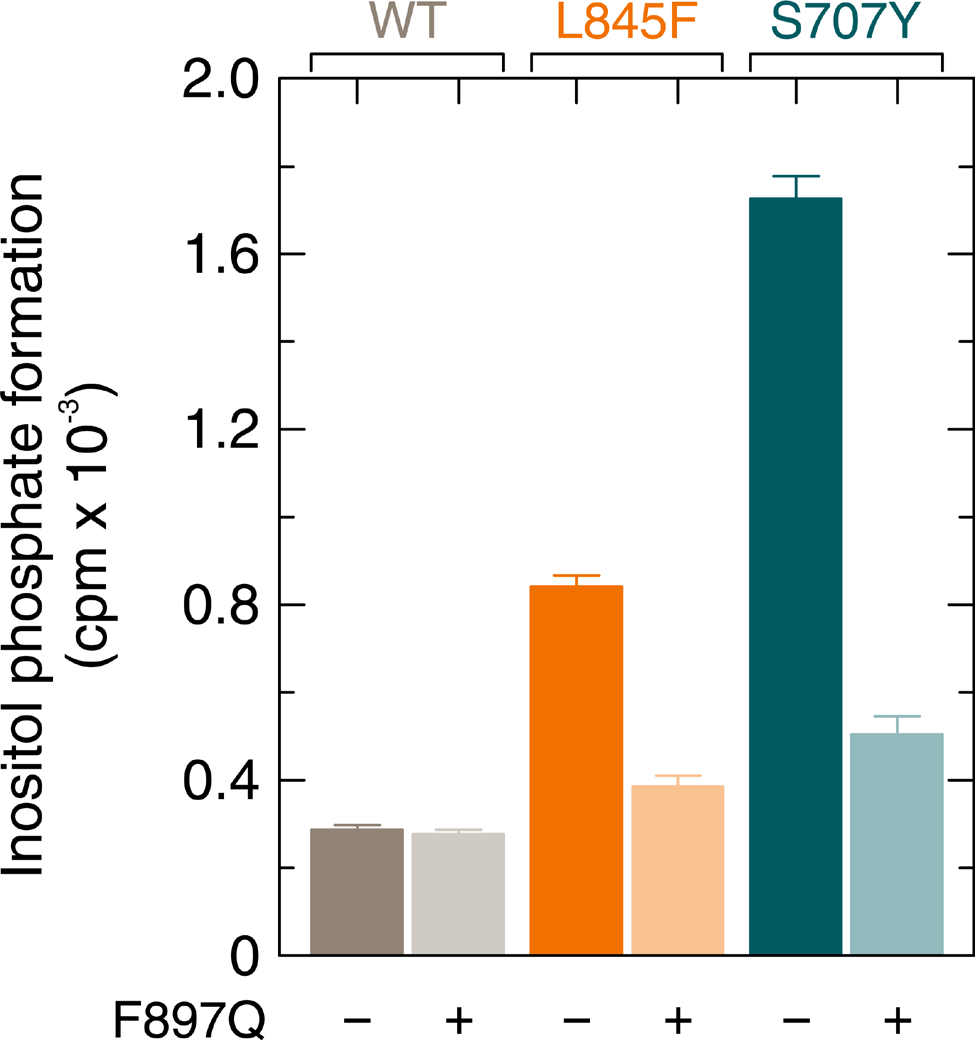
Enhanced Rac-stimulated activity of PLCγ_2_S707Y is prevented by a point mutation of PLCγ_2_, F897Q, mediating resistance of the enzyme to stimulation by activated Rac COS-7 cells were transfected with 150 ng/well of vector encoding either wild-type PLCγ_2_, PLCγ_2_F897Q, PLCγ_2_L845F, PLCγ_2_L845F/F897Q, PLCγ_2_S707Y, or PLCγ_2_S707Y/F897Q. Twenty four hours after transfection, the cells were incubated for 18 h with *myo*-[2-^3^H] inositol, and inositol phosphate formation was determined.

The F897Q substitution within PLCγ_2_ blocks activation of the enzyme by constitutively active Rac2^G12V^ and abolishes binding of GTPγS-activated Rac2 to PLCγ_2_ spPH but does not affect the overall fold of PLCγ_2_ spPH [18]. Figure 5 shows that the F897Q mutation caused similar reductions of basal activity of the L845F and S707Y variants of PLCγ_2_ (54% and 71%, respectively). This indicates that the basal activities of both mutants are strongly dependent on Rac1 endogenously present in COS-7 cells, similar to the activities of mutants R665W and L845F [17]. These reductions in PLCγ_2_ activity were not related to reduced synthesis of the F897Q single and compound PLCγ_2_ mutant proteins in transfected cells (Supplementary Figure 5).

### Basal activity of wild-type and S707Y mutant PLCγ_2_ is independent of protein phosphorylation at position 707

**Figure 6:**
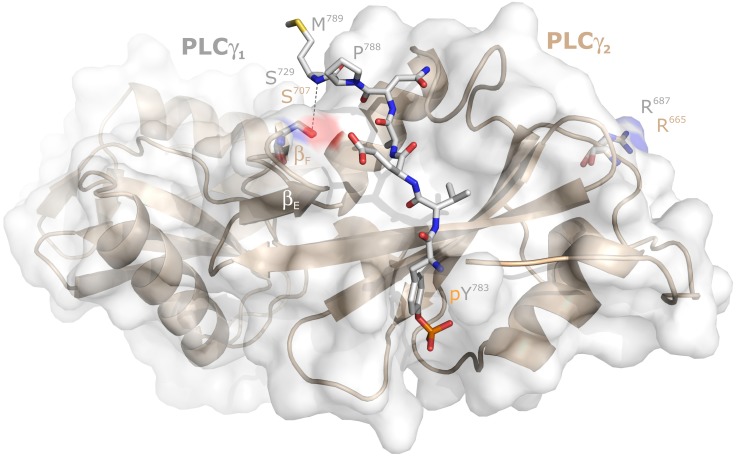
Localization of the PLCγ_2_ residue S707 relative to its PLC_γ1_ counterpart S729 in the intramolecular complex between the PLC_γ1_ SH2n-SH2c-tandem and its tyrosine phosphorylated (pY783) peptide between G781 and M789 The surface topology of PLCγ_1_ is shown as translucent gray surfaces. The structure of the SH2n-SH2c-tandem of PLCγ_2_, as predicted by Swiss-Model using the PLCγ_1_ counterpart as a template, is presented as wheat ribbons. The positions of S729 of PLCγ_1_ and S707 of PLCγ_2_, as well as those of R687 of PLCγ_1_ and R665 of PLCγ_2_, are almost superimposable and are shown as light blue and wheat sticks, respectively. The distance between the O_γ_ of S729 of PLCγ_1_ (4.3 Å) to the main chain NH of M789 of the PLCγ_1_ peptide forming an intramolecular interaction with the canonical SH2c domain pY binding pocket is shown as gray dashed line.

**Figure 7:**
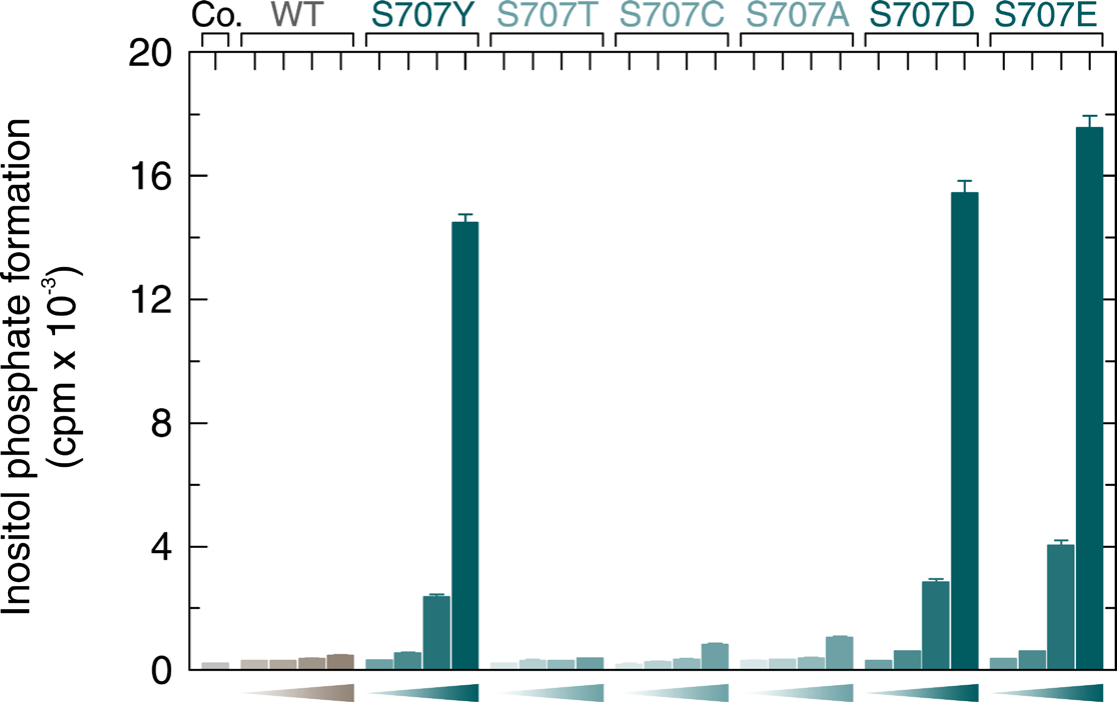
Basal activity of wild-type and S707Y mutant PLCγ_2_ is independent of protein phosphorylation at position 707 COS-7 cells were transfected as indicated with 500 ng/well of either empty vector (*Co.*, control) or increasing amounts (15, 50, 150, and 500 ng/well) of vector encoding either wild-type PLCγ_2_ (*WT*), PLCγ_2_S707Y (*S707Y*), PLCγ_2_S707T (*S707T*), PLCγ_2_S707C (*S707C*), PLCγ_2_S707A (*S707A*), PLCγ_2_S707D (*S707D*), or PLCγ_2_S707E (*S707E*). Twenty four hours after transfection, the cells were incubated for 18 h with *myo*-[2-^3^H]inositol, and inositol phosphate formation was then determined.

The next experiment was designed to address the question whether the stimulatory effects of mutations in position 707 of PLCγ_2_ were due to a specific loss of the serine residue at this position. This residue is shared in this position between PLCγ_1_ and PLCγ_2_ since the divergence of chondricthyes (cartilaginous fishes) from the ancestral vertebrate line about 528 million years ago [19], since it is already present in both PLCγ isoforms in the Australian ghostshark, *Callorhinchus milii* (acc. nos. XP_007903384.1 and XP_007887457.1, respectively). In human PLCγ_1_, the residue corresponding to S707 of PLCγ_2_, S729, is located at the beginning of β-strand F and its O_γ_ is in close distance (4.3 Å) to the main chain NH of M789 of a PLCγ_1_ peptide forming an intramolecular interaction with the cSH2 domain upon phosphorylation of its tyrosine residue Y783 (*cf.* Figure 6 and [20, 21]). To determine the requirement of the S707 side chain, S707 was replaced by either a threonine or a cysteine residue, resembling serine in some of their characteristics, or by an alanine to examine the importance of the polar side chains of serine, threonine, and cysteine altogether. The substitutions S707C and S707A were also selected to examine the possibility that S707 or T707 are substrates for a putative inhibitory protein serine or threonine phosphorylation. PLCγ_2_ can be heavily phosphorylated in B cells on unidentified serine residue(s) [22] and S707 is predicted *in silico* using online tools [23] to be phosphorylated by several protein kinases. Figure 7 shows, on the one hand, that the mutants S707T, S707C, and S707A were similar to wild-type PLCγ_2_ in their activities at increasing cellular expression levels. The latter three mutants and wild-type PLCγ_2_ were uniformly activated by Rac2^G12V^ (not shown), demonstrating that the mutants S707T, S707C, and S707A were in fact catalytically competent. On the other hand, the phosphomimetic mutants S707D and S707E resembled the mutant S707Y, further arguing against the notion that introduction of negative charges at S707 *via* constitutive serine phosphorylation would be responsible for the low basal activity of wild-type PLCγ_2_. Collectively, these results suggest that S707 can be replaced by several small amino acids to maintain low basal activity of PLCγ_2_, even by nonpolar residues not subject to protein phosphorylation such as alanine. Replacement by larger residues, such as Y, F, P, D, and E leads to increased basal activities of PLCγ_2_ in intact cells, irrespective of the polarity of the side chain. Phosphorylation of Y707 in the mutant S707Y, suggested earlier to be a potential cause of PLCγ_2_ activation [3, 24] is an unlikely reason of PLCγ_2_ activation, since the mutant S707F also displayed enhanced activity (*cf.* Figure 1B). Supplementary Figure 6 shows that the functional differences between the PLCγ_2_ variants observed in Figure 7 were not due to differences in protein expression.

### The co-occurrence of the mutations R665W, L845F, and S707Y provides synergistic effects to enhanced basal activity of PLCγ_2_

**Figure 8:**
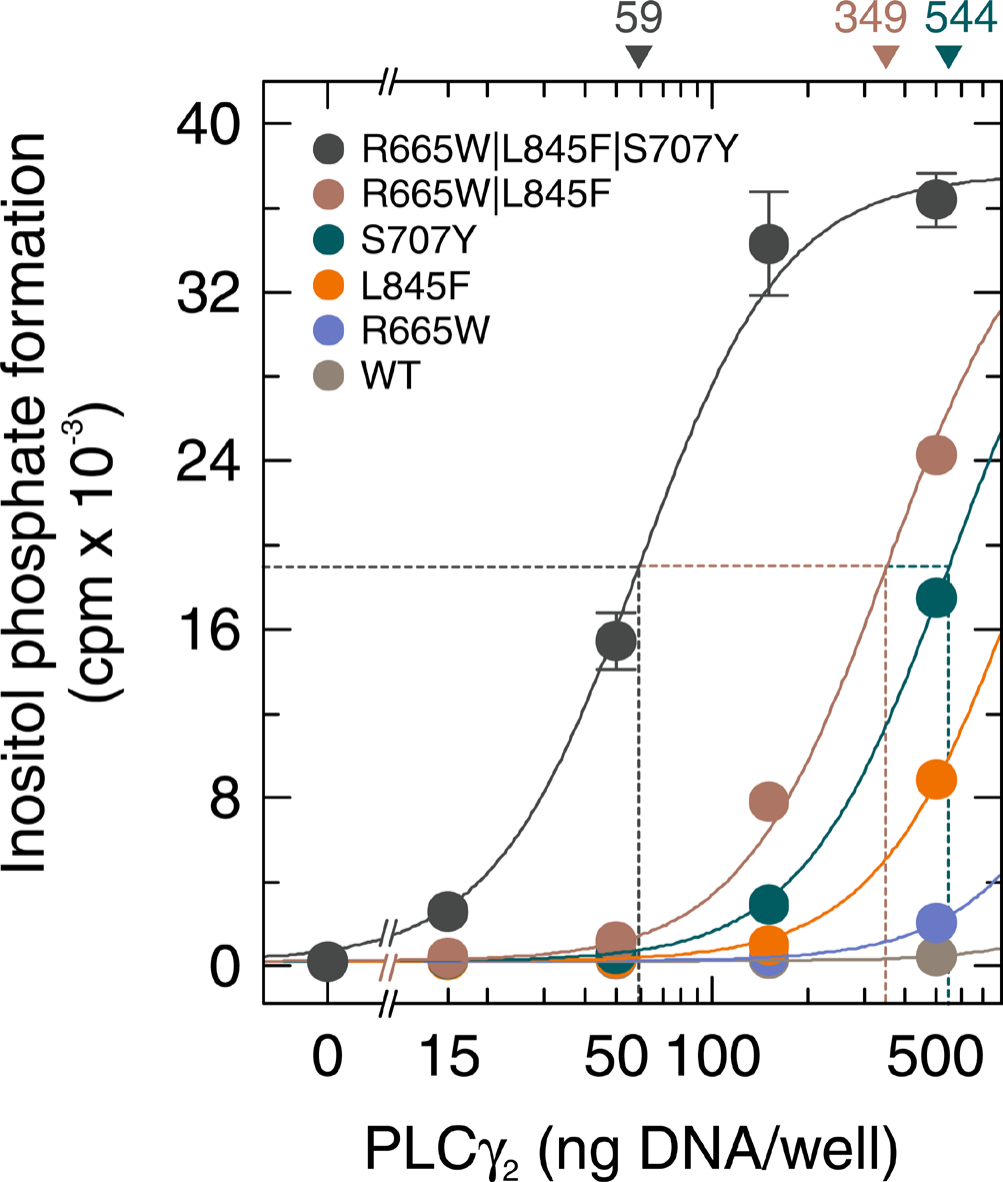
Mutations in *PLCG2* R665W, L845F, and S707Y synergize to promote enhancement of basal PLCγ_2_ activity COS-7 cells were transfected as indicated with increasing amounts (15, 50, 150, and 500 ng/well) of vector encoding either wild-type PLCγ_2_ (*WT*), PLCγ_2_R665W (*R665W*), PLCγ_2_L845F (*L845F*), PLCγ_2_S707Y (*S707Y*), PLCγ_2_R665W/L845F (*R665W/L845F*), or PLCγ_2_R665W/L845F/S707Y (*R665W/L845F/S707Y*). Twenty four hours after transfection, the cells were incubated for 18 h with *myo*-[2-^3^H]inositol, and inositol phosphate formation was then determined.

Multiple different *PLCG2* mutations co-occur in some patients and have been suggested to co-exist even in the same cell, at least in certain cases [7]. Specifically, patients exhibiting ibrutinib resistance have been identified with mutations co-occurring in their CLL cells in positions R665 and S707, in positions R665 and L845, in positions S707 and L845, as well as in positions R665, L845, and S707 [7]. We therefore examined the effect of a cumulative co-occurrence of these mutations on inositol phosphate formation by PLCγ_2_. Figure 8 shows that mutations R665W, L845F, and S707Y synergized to enhance basal activity in intact cells. For example, at 50 ng plasmid DNA/well, the activities of both compound mutants, R665W|L845F and R665W|L845F|S707Y, exceeded the combined activities of the constituent mutants (R665W, L845F, and S707Y) with high statistical significance (*P* < 0.001). This indicates that individual PLCγ_2_ mutants may be subject to further functional alteration by co-occurring mutations. Supplementary Figure 7 demonstrates that the functional differences observed in Figure 8 were not due to differences in protein expression of the PLCγ_2_ variants.

### Deletions and point mutations of *PLCG2* at residues S707 and A708 take different effects on enhanced basal PLCγ_2_ activity in intact cells

**Figure 9:**
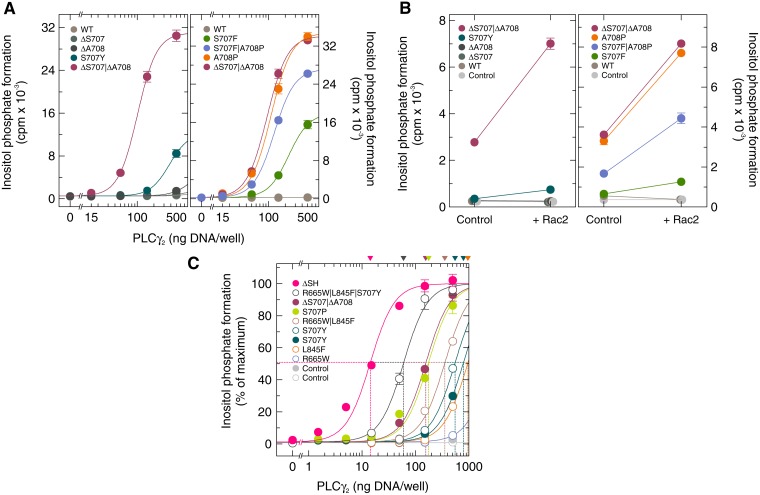
Deletions and mutations in *PLCG2* corresponding to residues S707 and A708 cooperate to promote enhancement of basal PLCγ_2_ activity and augment the responsiveness of the enzyme to wild-type Rac2 (**A**) COS-7 cells were transfected as indicated with increasing amounts (15, 50, 150, and 500 ng/well) of vector encoding either wild-type PLCγ_2_ (*WT*), PLCγ_2_ΔS707 (Δ*S707*), PLCγ_2_ΔA708 (Δ*A708*), PLCγ_2_ΔS707/ΔA708 (Δ*S707/*Δ*A708*), or PLCγ_2_S707Y (*S707Y*) (left panel). In the right panel, COS-7 cells were transfected with vector encoding either wild-type PLCγ_2_ (*WT*), PLCγ_2_S707F (*S707F*), PLCγ_2_A708P (*A708P*), PLCγ_2_S707F/A708P (*S707F/A708P*), or PLCγ_2_ΔS707/ΔA708 (Δ*S707/*Δ*A708*). Twenty four hours after transfection, the cells were incubated for 18 h with *myo*-[2-^3^H]inositol, and inositol phosphate formation was then determined. (**B**) COS-7 cells were transfected as indicated with empty vector (*Control*) or 50 ng/well of vector encoding wild-type PLCγ_2_ (*WT*), PLCγ_2_ΔS707 (Δ*S707*), PLCγ_2_ΔA708 (Δ*A708*), PLCγ_2_ΔS707/ΔA708 (Δ*S707/*Δ*A708*), or PLCγ_2_S707Y (*S707Y*) (left panel) or with 50 ng/well of vector encoding wild-type PLCγ_2_ (*WT*), PLCγ_2_S707F (*S707F*), PLCγ_2_A708P (*A708P*), PLCγ_2_S707F/A708P (*S707F/A708P*), or PLCγ_2_ΔS707/ΔA708 (Δ*S707/*Δ*A708*) (right panel), and 25 ng/well of vector encoding wild-type Rac2. (**C**) none of the disease-related PLCγ_2_ point mutations leads to a complete loss of nSH2-cSH2-SH3-mediated autoinhibition. COS-7 cells were transfected with 500 ng of either empty vector (*Control*) or increasing amounts (1.5, 5, 15, 50, 150, and 500 ng/well) of vector encoding either PLCγ_2_S707Y (*S707Y*), PLCγ_2_S707P (*S707P*), PLCγ_2_ΔS707/ΔA708 (Δ*S707/*Δ*A708*), or PLCγ_2_ΔSH (Δ*SH*) (*closed symbols*). The *open symbols* and the *corresponding curves* are replotted from Figure 8.

Very recently, two CLL patients progressing on ibrutinib have been described carrying a 6-nucleotide deletion in exon 20 of *PLCG2* (c.2120-2125del), leading to the deletion of both S707 and A708 [8, 9]. In addition, two patients were found with a A708P point mutation and one with a S707F|A708P double mutation [7]. We therefore set out to determine the effects of these mutations, alone or in combination, on basal activity of PLCγ_2_ in intact cells. Figure 9A, left panel, illustrates that there were relatively-small-to-moderate, dose-dependent increases of inositol phosphate formation with increasing amounts of PLCγ_2_-encoding DNA, which were ~1.7-fold and ~4.7-fold, relative to WT PLCγ_2_, for the mutants ΔS707 and ΔA708, respectively. The degree of this effect was augmented to ~22-fold for the double mutant ΔS707|ΔA708, even higher than for the mutant S707Y (~9.0-fold). Thus, the two deletions, ΔS707 and ΔA708, clearly have the potential to synergistically enhance PLCγ_2_ activity in ibrutinib-resistant CLL cells. A different pattern was observed for the point mutations in positions S707 and A708 (Figure 9A, right panel). In this case, maximal inositol phosphate formation was observed for the single mutant A708P (~146-fold relative to wild-type PLCγ_2_). This activity was similar to that of the ΔS707|ΔA708 double mutant, but clearly higher than both the S707F single mutant (~75-fold) and, even more interestingly, the double mutant S707F|A708P (~114-fold). Hence, the cumulative stimulatory effect of individual PLCγ_2_ point mutations on inositol phosphate formation may be observed in some, but not all cases. As shown in Supplementary Figure 8A, the PLCγ_2_ variants examined in Figure 9A were similarly expressed in transfected cells. Figure 9B illustrates that the increases in basal inositol phosphate formation by the mutants shown in Figure 9A correlated closely with the ability of exogenous wild-type Rac2 to further enhance this activity. Specifically, the order of the PLCγ_2_ mutants in regard to this enhancement was: ΔS707|ΔA708 >> S707Y > ΔA708 > ΔS707 and A708P > S707F|A708P > S707F. There was no major change in PLCγ_2_ protein expression upon co-expression of Rac2 (Supplementary Figure 8B).

Next, we compared the effects of *PLCG2* mutations observed in ibrutinib-resistant CLL patients to a *PLCG2* deletion, ΔSH, effectively removing the entire autoinhibitory nSH2-cSH2-SH3 region of the encoded protein and previously characterized as one of the most active deletion mutant of PLCγ [16, 25]. Figure 9C shows that the disease-related PLCγ_2_ point mutants showed marked differences in their activities, but that none of them reached the high activity of PLCγ_2_ΔSH, which was still 4.2-fold higher (based on the amounts of cDNA required for half-maximal inositol phosphate formation) than the activity of PLCγ_2_R665W|L845F|S707Y, the most active of the point mutants mediating ibrutinib resistance in CLL patients. Overall, as judged by the apparent ED_50_ values determined or extrapolated in this experiment, there was an about 100-fold difference between PLCγ_2_ΔSH and the PLCγ_2_ mutant with the lowest activity, R665W. Supplementary Figure 8C shows that the different activities of the PLCγ_2_ variants shown in Figure 9C were unlikely to be caused by differences in protein expression. Thus, inasmuch as the individual ibrutinib resistance mutation relieve PLCγ_2_ from an autoinhibitory constraint, none of the disease-related point mutations examined here results in a complete loss of nSH2-cSH2-SH3-mediated autoinhibition.

### PLCγ_2_S707Y is not constitutively active in a cell-free system employing artificial lipid vesicles

**Figure 10:**
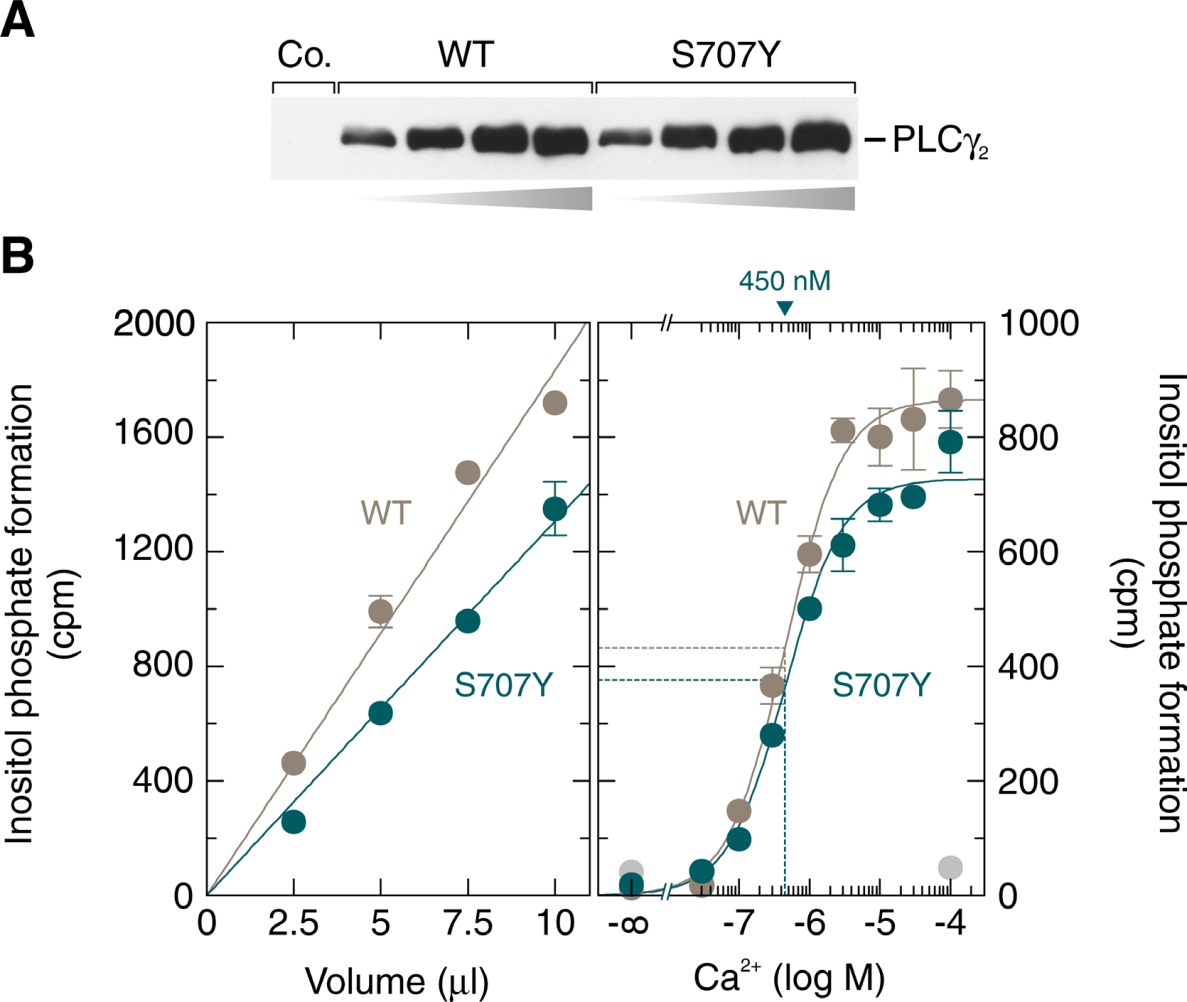
The activities of soluble, insect-cell-expressed wild-type and S707Y mutant PLCγ_2_ in a cell-free system made up of artificial lipid vesicles are similar (**A**) Aliquots of soluble fractions of Sf9 cells infected with baculovirus encoding either β-galactosidase (*Co.*, control), wild-type or S707Y mutant PLCγ_2_ that had been adjusted to contain equal amounts of recombinant PLCγ_2_ were subjected in increasing amounts of total protein to SDS-PAGE and immunoblotting was performed using antibodies reactive against the c-Myc epitope. (**B**) aliquots of the two samples analyzed in (A) containing equal quantities of wild-type or S707Y mutant PLCγ_2_ were incubated for 45 min at 30° C at 10 μM free Ca^2+^ in the presence of 2.5 mM sodium deoxycholate with phospholipid vesicles containing [^3^H]PtdIns*P*_2_ (left panel). In the right panel, aliquots of the two samples analyzed in (A) containing equal quantities of wild-type or S707Y mutant PLCγ_2_ were incubated at increasing concentrations of free Ca^2+^ and 2.5 mM sodium deoxycholate with phospholipid vesicles containing [^3^H]PtdIns*P*_2_. The EC_50_ values of Ca^2+^ for the stimulation of wild-type or S707Y mutant PLCγ_2_ activity obtained by non-linear curve fitting is shown *above the graphs* in nanomolar.

To characterize the functional role of residue S707 at the biochemical level, wild-type and S707Y mutant PLCγ_2_ were produced as recombinant, soluble polypeptides in baculovirus-infected insect cells and assayed for their ability to hydrolyze [^3^H]PtdIns*P*_2_ in a cell-free system employing artificial lipid vesicles. Samples containing either wild-type or S707Y mutant PLCγ_2_ were first adjusted by semi-quantitative immunoblotting to contain similar amounts of recombinant PLCγ_2_ protein (Figure 10A). Aliquots containing equal quantities of either wild-type or S707Y mutant PLCγ_2_ were then either assayed for PLC activity at 10 μM free Ca^2+^ and 2.5 mM sodium deoxycholate at increasing amounts of protein (Figure 10B, left panel) or at a fixed amount of protein, 2.5 mM sodium deoxycholate, and increasing concentrations of free Ca^2+^ (Figure 10B, right panel). The results show that the activities of both PLCγ_2_ isozymes increased linearly with increasing protein and displayed similar dependencies on free Ca^2+^, with half-maximal and maximal hydrolysis occurring at ~450 nM and ~10 μM free Ca^2+^, respectively. More importantly and much to our surprise, the activities of the S707Y mutant of PLCγ_2_ were even lower, by ~29 % and ~12 % (Figure 10B, left and right panels, respectively), than those of the wild-type enzyme. Thus, the striking activation of the mutant enzyme observed in intact cells (*cf.*, e.g., Figure 1) is not detected when the enzyme is reconstituted *in vitro* with its substrate in lipid vesicles.

### PLCγ_2_S707Y is hypersensitive to EGF stimulation mediated by both tyrosine phosphorylation of the enzyme and its activation by Rac

**Figure 11:**
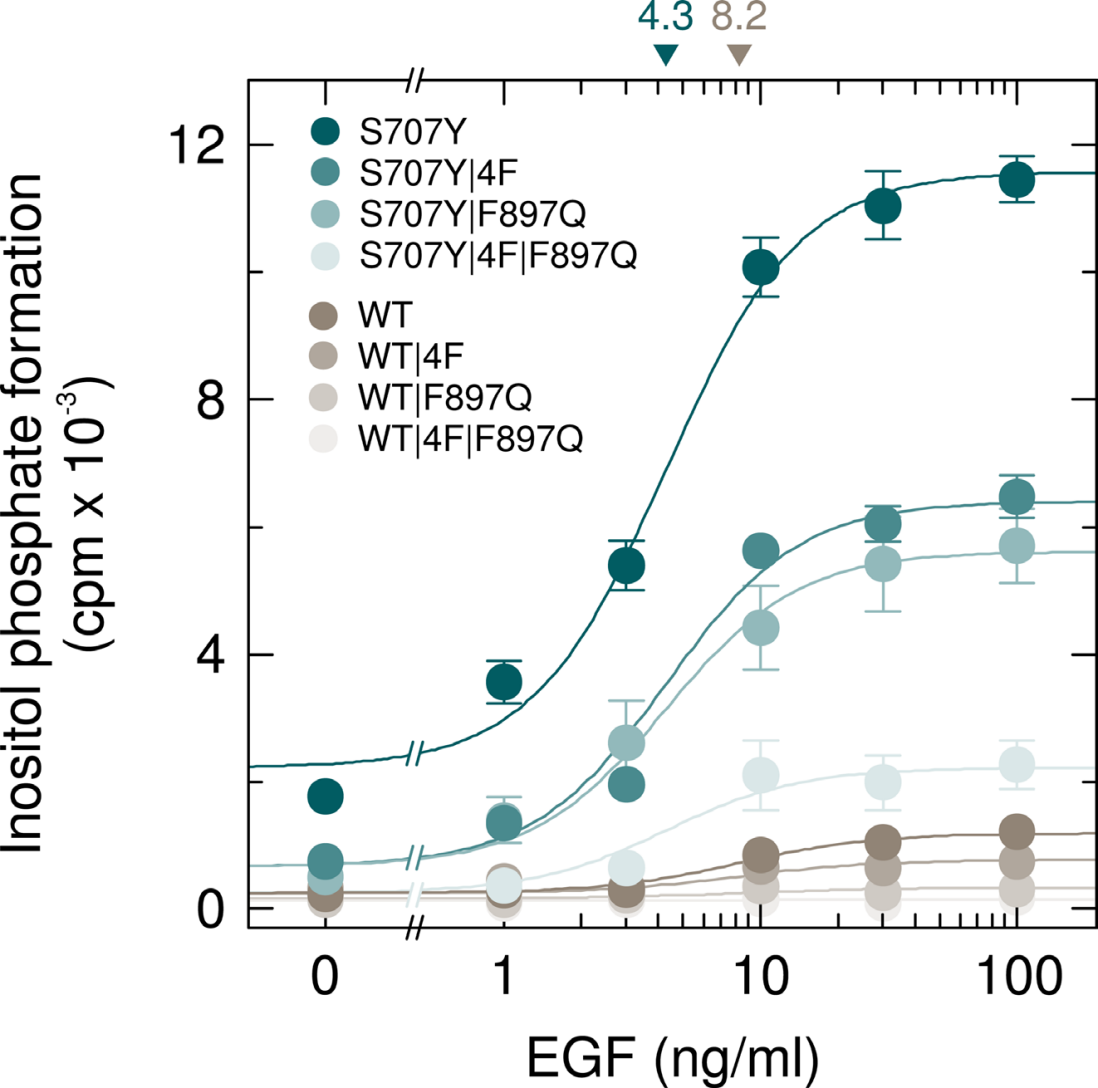
PLCγ_2_S707Y is hypersensitive to activation by EGF receptor(s) endogenously expressed in COS-7 cells by a mechanism dependent on both protein tyrosine phosphorylation and activation by Rac COS-7 cells were transfected with 150 ng/well of vectors encoding either PLCγ_2_S707Y (*S707Y*), PLCγ_2_S707Y/4F (*S707Y*/*4F*), PLCγ_2_S707Y/F897Q (*S707Y*/*F897Q*), or PLCγ_2_S707Y/4F/F897Q (*S707Y*/*4F*/*F897Q*) or wild-type PLCγ_2_ (*WT*), PLCγ_2_4F (*WT/4F*), PLCγ_2_F897Q (*WT*/*F897Q*), or PLCγ_2_4F/F897Q (*WT*/*4F*/*F897Q*) (all constructed in pMT2 vector; 4F, Tyr to Phe substitutions at amino acid positions 753, 759, 1197, and 1217). Eighteen hours after transfection, the cells were incubated for a further 24 h with *myo*-[2-^3^H]inositol in the absence of serum and then treated for 60 min in the presence of 20 mM LiCl with increasing concentrations of EGF (1, 3, 10, 30, and 100 ng/ml), followed by determination of inositol phosphate formation. Background inositol phosphate formation in response to addition of EGF at increasing concentrations was determined in parallel on cells transfected with empty vector and subtracted from the individual values, with appropriate consideration of error propagation [47]. The EC_50_ values of EGF for the stimulation of wild-type or S707Y mutant PLCγ_2_ activity obtained by non-linear curve fitting are shown *above the graphs* in nanograms/ml.

COS-7 cells exhibit endogenous expression of EGF receptors, known to be capable of causing activation of exogenous PLCγ_2_ by both protein tyrosine phosphorylation and activation of Rac [17]. Figure 11 shows a comparison of the activation of wild-type and S707Y mutant PLCγ_2_ by EGF and an analysis of the relative contribution of intramolecular tyrosine-phosphorylation-mediated and Rac-mediated activation. The latter was done by studying the two PLCγ_2_ isoforms carrying either phenylalanine replacements of the four tyrosines known to be phosphorylated by upstream tyrosine kinases during enzyme activation (Y753, Y759, Y1197, Y1217; 4F) or the F897Q mutation conferring Rac resistance to PLCγ_2_. There was a concentration-dependent increase of wild-type and S707Y mutant PLCγ_2_ stimulation by EGF, which was half-maximal at ~8.2 ng/ml and ~4.3 ng/ml EGF, respectively, and maximal at ~30 ng/ml. Maximal EGF-stimulated PLCγ_2_ activity was ~9.7-fold higher in the presence of PLCγ_2_S707Y in comparison with wild-type PLCγ_2_. The results obtained with the 4F, F897Q, and 4F/F897Q mutants of the two variants suggested that similar proportions of a about 40 % to 50 % of the responses of both wild-type and S707Y mutant PLCγ_2_ were due to intramolecular tyrosine-phosphorylation- and Rac-mediated activation, respectively. Note that PLCγ_2_S707Y was sensitive to activation by EGF even in the presence of both the 4F and F897Q mutations. Supplementary Figure 9 shows that wild-type and mutant PLCγ_2_ isozymes were present at equal amounts throughout the experiment presented in Figure 11. It is clear from the results shown in Figure 11 and those shown in Figure 2–5 that the stimulatory effect of the S707Y mutation synergizes with the stimulatory effects of both of the regulatory inputs into PLCγ_2_, protein tyrosine phosphorylation (Figure 11) and Rac (Figures 2–5).

### Unlike the two PLAID PLCγ_2_ mutants Δ19 and Δ20-22, the APLAID and ibrutinib resistance mutant PLCγ_2_S707Y is not activated by cold temperatures

**Figure 12:**
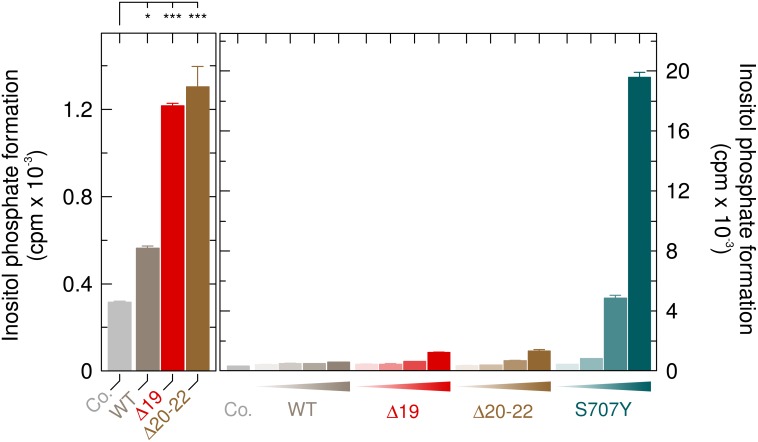
Comparison of the APLAID point mutant PLCγ_2_S707Y to the PLAID deletion mutants PLCγ_2_Δ19 and PLCγ_2_Δ20-22 at 37° C Left panel, COS-7 cells were transfected as indicated with 500 ng/well of either empty vector (*Co.*, control) or vector encoding either wild-type PLCγ_2_ (*WT*), PLCγ_2_Δ19 (Δ*19*) or PLCγ_2_Δ20-22 (Δ*20-22*). Right panel, COS-7 cells were transfected as indicated with 500 ng/well of either empty vector (*Co.*, control) or increasing amounts (15, 50, 150, and 500 ng DNA/well) of vector encoding either wild-type PLCγ_2_ (*WT*), PLCγ_2_Δ19 (Δ*19*), PLCγ_2_Δ20-22 (Δ*20-22*), or PLCγ_2_S707Y (*S707Y*). In the left panel, the data was analyzed with One-way Analysis of Variance (ANOVA) with Bonferroni Multiple Comparisons Test contained in the GraphPad InStat software package (version 3.10; GraphPad Software, La Jolla, CA). Statistically significant effects are denoted by ^***^P < 0.001; and ^*^, 0.01 < *P* < 0.05.

Since the *PLCG2* mutation causing the S707Y substitution also provides the molecular basis of the germline monogenic autoinflammatory disorder APLAID, we compared the activity of PLCγ_2_S707Y to those of the two mutants causing the related disease PLAID, PLCγ_2_Δ19 and PLCγ_2_Δ20-22. Figure 12, left panel, shows that wild-type PLCγ_2_ and its mutants PLCγ_2_Δ19 and PLCγ_2_Δ20-22 caused only modest enhancements of inositol phosphate formation ranging from ~ 1.8-fold to ~ 4.1-fold even at the highest expression levels. In marked contrast (Figure 12, right panel), enhanced PLC activity by PLCγ_2_S707Y was already evident at the lowest expression level (~1.3-fold) and amounted to approximately 62-fold at the highest expression level. Supplementary Figure 10 shows that there were only minor, if any differences in protein expression between the PLCγ_2_ variants tested in Figure 12.

**Figure 13:**
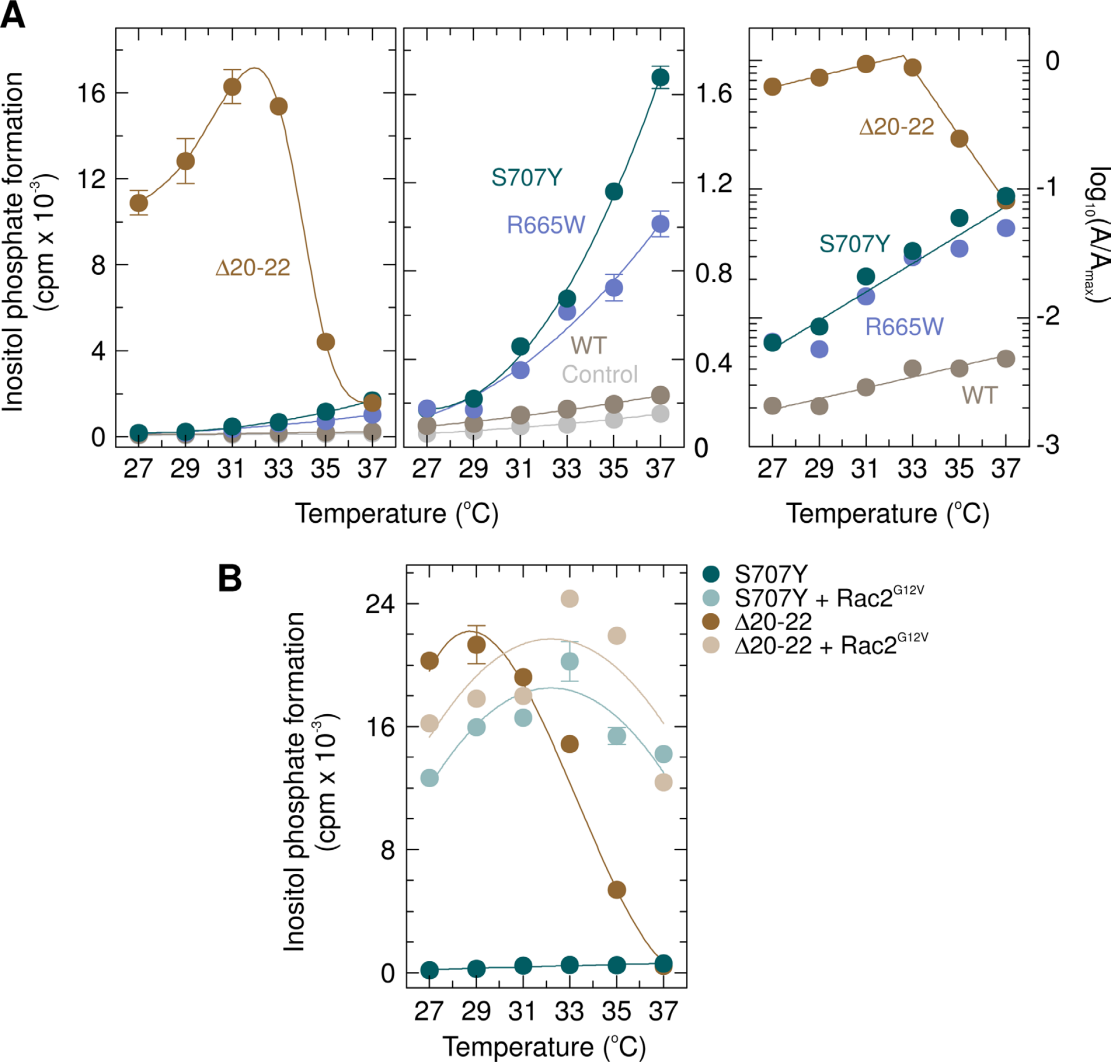
Unlike the PLAID PLCγ_2_ deletion mutant PLC_γ_2_Δ_20–22, neither APLAID and ibrutinib resistance PLCγ_2_ mutant S707Y nor the ibrutinib resistance PLCγ_2_ mutant R665W are activated by cool temperatures (**A**) left panel, COS-7 cells were transfected with empty vector (*Control*) or 500 ng/well of vector encoding either wild-type PLCγ_2_ (*WT*) or PLCγ_2_R665W (*R665W*), or 100 ng/well of vector encoding either PLCγ_2_S707Y (*S707Y*) or PLCγ_2_Δ20-22 (Δ*20-22*). Twenty-four hours after transfection, the cells were incubated for 18 h with *myo*-[2-^3^H]inositol at the indicated temperatures and inositol phosphate formation was then determined. The levels of inositol phosphate formation in control cells (*Control*) and cells expressing wild-type PLCγ_2_ (*WT*), PLCγ_2_R665W (*R665W*), or PLCγ_2_S707Y (*S707Y*) are shown in expanded scale on the center panel. Right panel, determination of the 10-degree temperature coefficients, *Q*_10_. The data shown in *panel A* on the cool temperature responses of cells expressing wild-type PLCγ_2_ (*WT*), PLCγ_2_Δ20–22 (Δ*20–22*), PLCγ_2_R665W (*R665W*), and PLCγ_2_S707Y (*S707Y*) was taken to determine the Q_10_ values of these responses as detailed in Experimental Procedures. The individual temperatures *T_i_* were plotted against, with the interpolated maximum activity of PLCγ_2_Δ20-22 at 32° C chosen as the reference activity *A_ref_* and reference temperature, *T_ref_*, respectively. The data of the linear components was analyzed by non-linear least square curve fitting to a polynominal first order (straight line) equation. The slope of the curve of PLCγ_2_Δ20–22 between 27° C and 31° C was not significantly different by global curve fitting from the slope obtained for wild-type PLCγ_2_ (*P* = 0.9356 and *P* = 0.2121, respectively). The slopes of the curves of PLCγ_2_S707Y and PLCγ_2_R665W were not significantly different by global curve fitting from each other but different from the slope obtained for PLCγ_2_Δ20–22 between 27° C and 31° C and wild-type PLCγ_2_ (*P* = 0.9356 and *P* = 0.2121, respectively). (**B**) Unlike PLCγ_2_Δ20–22, where a decrease in the incubation temperature resulted in a progressive loss of the Rac2^G12V^ stimulatory activity, the mutant S707Y is also stimulated by Rac2^G12V^ at low temperatures. COS-7 cells were transfected with 50 ng/well of vector encoding either PLCγ_2_S707Y (*S707Y*) or PLCγ_2_Δ20–22 (Δ*20–22*) and 25 ng/well of empty vector or vector encoding Rac2^G12V^. Twenty four hours after transfection, the cells were incubated for 18 h with *myo*-[2-^3^H]inositol at the indicated temperatures and inositol phosphate formation was then determined.

A comparison of the response of the PLCγ_2_ PLAID mutant Δ20–22 and the mutants S707Y and R665W as well as wild-type PLCγ_2_ to cooling from 37° C to 27° C is shown in Figure 13. As reported earlier, inositol phosphate formation was markedly (~11-fold) enhanced when cells expressing PLCγ_2_Δ20–22 were incubated at temperatures only slightly lower than 37° C. There was a biphasic stimulatory response with declining temperatures, with a maximum at ~32° C and a gradual reduction upon further cooling to 27° C. Thus, at ~32° C, the absolute increase in inositol phosphate formation in cells expressing PLCγ_2_Δ20–22 over basal activity of mock-transfected control cells was enhanced about 260-fold relative to the increase over basal inositol phosphate formation observed in cells expressing wild-type PLCγ_2_. In marked contrast, the responses of cells containing any of the other PLCγ_2_ isozymes and of control cells displayed only a single, decreasing phase of inositol phosphate formation, when the temperature was reduced from 37° C to 27° C (Figure 13A, left and center panels). The determination of the 10° C temperature coefficient (*Q*_10_) values for the mutants Δ20–22, S707Y, and R665W, as well as wild-type PLCγ_2_ to cooling from 37° C to 27° C is shown in Figure 13A, right panel. While PLCγ_2_Δ20–22 displayed two separate, linear phases of opposite signs and markedly distinct *Q*_10_ values (2.74 and 382, respectively), only a single linear component with a *Q*_10_ value of 2.74 was evident for wild-type PLCγ_2_ between 37° C and 27° C. Single component, linear responses were also observed for the PLCγ_2_ mutants S707Y and R665W, with identical *Q*_10_ values (12.8), i.e. ~4.7-fold higher than those of the wild-type enzyme and the PLAID mutant at lower temperatures. The experiment shown in Figure 13B was carried out to determine the effect of decreasing temperatures on the ability of constitutively active Rac2^G12V^ to activate PLCγ_2_S707Y in comparison to PLCγ_2_Δ20-22. We have shown earlier that cooling caused progressive loss of the stimulatory effect of Rac2^G12V^ on PLCγ_2_Δ19, but not on wild-type PLCγ_2_ [16]. Figure 13B shows that at 37° C, Rac2^G12V^ caused similar enhancements of the activity of PLCγ_2_S707Y and PLCγ_2_Δ20-22 (~ 21-fold and ~27-fold, respectively). Lowering the incubation temperature, however, took a very different effect on Rac2^G12V^-mediated activation of the two enzyme variants. While PLCγ_2_Δ20-22 gradually lost stimulation by Rac2^G12V^, the stimulatory effect of Rac2^G12V^ on PLCγ_2_S707Y was maintained upon cooling, such that the degree of activation was even enhanced to ~60-fold at 27° C. As shown previously, loss of Rac stimulation of PLAID PLCγ_2_ mutants is unlikely to be due to exhaustion of the inositol phospholipids substrate at lower temperatures [16]. Thus, except for the higher PLC activities over the whole range of temperatures from 37° C to 27° C (Figure 13A, center panel), PLCγ_2_S707Y resembles wild-type PLCγ_2_ [16] with regard to the effect of cooling on its activation by Rac2^G12V^. Supplementary Figure 11 shows that the functional changes observed in Figure 13A and 13B were not explained by changes in mutant PLCγ_2_ or Rac2^G12V^ expression as a function of cooling.

**Figure 14:**
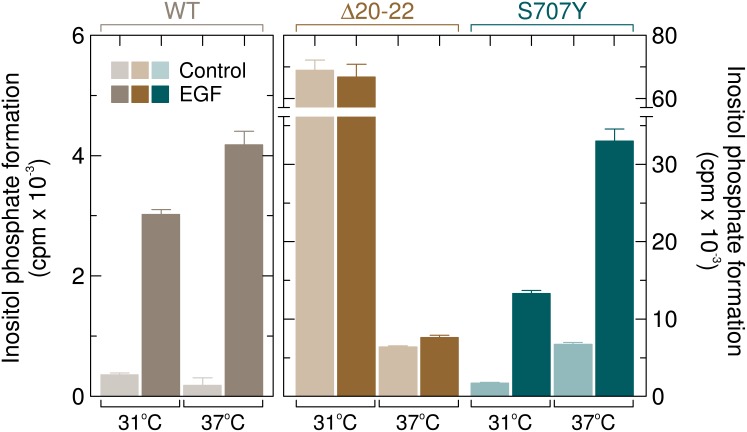
Unlike the PLCγ_2_ deletion mutant Δ20–22, PLCγ_2_S707Y is sensitive to stimulation by EGF COS-7 cells were transfected with vectors encoding either wild-type PLCγ_2_ (*WT*) (500 ng per well, left panel), PLCγ_2_Δ20–22 (Δ20–22) or PLCγ_2_S707Y (*S707Y*) (both at 150 ng per well). Eighteen hours after transfection, the cells were incubated for a further 24 h as indicated at either 31° C or 37° C with *myo*-[2-^3^H]inositol and 10 mM LiCl in the absence of serum and then treated for 60 min at the same temperatures in the presence of 10 mM LiCl with 100 ng/ml EGF, followed by determination of inositol phosphate formation. Background inositol phosphate formation in response to addition of EGF was determined in parallel on cells transfected with empty vector and subtracted from the individual values, with appropriate consideration of error propagation [47]. Additional experiments showed that the stimulatory effect of EGF on wild-type PLCγ_2_ activity was concentration-dependent with half-maximal and maximal effects at approximately 10 ng/ml and 50 ng/ml, respectively, and was almost completely blocked (~95%) by the EGFR inhibitor cetuximab (not shown).

Figure 14 shows that addition of 100 ng/ml of EGF to cells expressing wild-type or mutant PLCγ_2_ caused marked activation of wild-type PLCγ_2,_ which was higher at 37° C (~22-fold) than at 31° C (~8.5-fold). In contrast and as reported before [16], the activity of PLCγ_2_Δ20–22 was strongly (~13-fold) enhanced by cooling from 37° C to 31° C but was only marginally increased (~1.2-fold) or not affected by addition of EGF at 37° C and 31° C, respectively. Cells expressing the PLCγ_2_ mutant S707Y were very similar, in their response to EGF and cooling, to cells harboring wild-type PLCγ_2_, but clearly distinct from cells expressing PLCγ_2_Δ20-22. Thus, EGF enhanced inositol phosphate formation by ~7.7-fold at 31° C and by ~4.9-fold at 37° C. In relative terms (fold stimulation), the latter value was lower than the one observed for the wild-type enzyme, presumably due to enhanced basal activity of the mutant enzyme at 37° C. In absolute terms, however, the mutant S707Y showed a much (~5.3-fold) greater response to addition of EGF than its wild-type counterpart. Supplementary Figure 12 shows that there was no change in the abundance of the various PLCγ_2_ isoforms in response to addition of EGF.

## DISCUSSION

The results shown herein demonstrate that mutations in position S707 of PLCγ_2_ affect PLCγ_2_ activity and regulation in intact cells in a way that is qualitatively similar to the mutations R665W and L845F, although the structural changes occur in topologically distinct regions of the enzyme. Specifically, R665 and S707 are located on the opposite surfaces of the cSH2 domain in its predicted three-dimensional structure (*cf.* Figure 6), L845 resides at the beginning of the C-terminal half of the split PH domain. The functional consequences of the three mutations are different, however, in quantitative terms. Thus, the mutants S707Y, S707F, and S707P, all occurring in CLL cells of ibrutinib-resistant patients, display higher basal PLC activity in intact cells than the mutants R665W and L845F. A similar order (R665W < L845F < S707Y ~S707F < S707P) was observed, both in terms of the apparent efficacies and potencies, for the activation of the mutants by wild-type Rac2 upon co-expression in intact cells. This effect is presumably due to activation of Rac2 by active Rac guanine nucleotide exchange factors (GEFs) endogenously present in COS-7 cells [17]. On the basis of these results, it appears likely that resistance to ibrutinib of tumor cells harboring a *PLCG2* mutation is not an all-or-nothing, quantal process, but rather a graded response depending on both the site and the nature of the amino acid substitution within the PLCγ_2_ protein.

At first glance, the observation of a combinatorial enhancement of basal activity in response to more than one ibrutinib resistance mutation in a single PLCγ_2_ molecule, such as R665W, S707Y, and L845F suggests that the upper limit of a graded ibrutinib resistance in CLL patients may also be extended by the number of mutations co-occurring within a single *PLCG2* gene of these patients. However, residues R665, S707, and L845 are encoded by exons 19, 20, and 24 of the *PLCG2* gene and their codons are present in the gene at distances of ~6.9 kB (R665 to S707) and ~9.0 kB (S707 to L845). Given that the read lengths of next generation sequencing are typically < 700 bp for short-read approaches [26], it is unclear from sequencing of genomic DNA fragments whether the above *PLCG2* mutations, if identified in a single patient reside in a single *PLCG2* copy, in two alleles from the same CLL cell, or in distinct genes from distinct cell clones. Nevertheless, as shown in Figs. 8 and 9, if the mutations occur in a single gene, they clearly have the potential to synergize in regard to enhancing PLCγ_2_ activity in intact cells. This view is strongly supported by the experiment investigating the combinatorial functional effects of deletions in positions S707 and A708, both encoded by exon 20, although thus far only a deletion of both residues has been reported for ibrutinib-resistant patients [8, 9].

Although the exact number of genetic modifications to develop malignant tumors is not certain and may differ between different tumor types, it seems clear that the development of malignancies is a multistep process [27]. By the same token, resistance to anticancer therapies in the advanced setting may involve several (epi)genetic alterations, either in distinct or in the same genes or pathways, resulting in either parallel or convergent evolution of drug resistance [27]. Very interestingly, at least two studies have shown that the temporal order of genetic events influences cancer biology (reviewed in [28]).

With regard to the development of ibrutinib resistance in CLL patients, S707F point mutations have been detected in resistant cells without mutations in position A708 [5–7], in one case even in pretreatment samples before the initiation of targeted inhibition of Btk [6]. The A708P point mutation without alterations in S707 has been identified in two patients, the S707F/A708P double mutation in one patient [7]. Both S707 and A708 are encoded by exon 20 of *PLCG2*. Hence, the S707F/A708P double mutation must reside in a single *PLCG2* gene. In this case, however, the order of basal PLCγ_2_ activities in intact cells is A708P > S707F/A708P > S707F > WT (*cf.* Figure 9A). This indicates that, if mutations at the S707-A708 hot spot develop sequentially rather than simultaneously, the functional consequence of the mutations is dependent on their temporal order. If mutation A708P occurs before mutation S707F, then the latter mutation decreases basal inositol phosphate formation by PLCγ_2_ and, by inference, the extent of ibrutinib resistance, whereas an increase would be predicted for the opposite order. Thus, early genetic ibrutinib resistance mutations of *PLCG2* may affect the biological impact of subsequent mutations, extending the concept of “genetic canalization” [27] from tumorigenesis to development of resistance to targeted tumor drugs.

Although the exact three-dimensional structure of the PLCγ_2_ region encompassing residue S707 is unknown, the known structures of the corresponding region in PLCγ_1_, with peptides holding a phosphorylated tyrosine residue (2pld; 3gqi; 4ey0; 4k44; 5eg3) or a non-phosphorylated peptide counterpart (4fbn) can be used to obtain a model of its topology (*cf.* Figure 6). According to this model, S707 is located at the base of one end of an extended groove in cSH2 representing a binding site for a peptide sequence comprised of residues M758 to I765 from the linker between cSH2 and SH3. The latter peptide contains the protein tyrosine kinase substrate Y759, which is most homologous in terms of location and spacing to Y783 phosphorylated by upstream protein tyrosine kinases in PLCγ_1_. Similar to the orientation in PLCγ_1_ of the side chains of V784 (pY+1) and A786 (pY+3) into a shallow hydrophobic pocket formed by cSH2 residues F706, L726, L746, and Y747 [20], the side chains of PLCγ_2_ linker peptide residues V760 (pY+1) and P763 (pY+3) are predicted to be directed to a conserved apolar pocked formed by F684, L704, L725, and Y726 of PLCγ_2_. The pY759 binding pocket of PLCγ_2_ cSH2 is likely to be made up of four arginines (R653, R672, R674, and R694), which correspond to R675, R694, R696, and R716 of PLCγ_1_, constituting the binding pocket for pY783 in PLCγ_1_ cSH2.

The observation that charge-modifying replacements of N728, Y747/R748, R748, or R753 by glutamic acid caused activation of PLCγ_1_ led to the suggestion that these cSH2 residues are the main constituents of an electropositive interface of PLCγ_1_ cSH2. This interface was suggested to mediate PLCγ_1_ autoinhibition by interacting with electronegative residues on the catalytic core of PLCγ_1_, presumably D342, E347, and D1019, thus precluding its access to its membrane-associated phospholipids substrate [20, 21]. However, PLCγ_2_ contains a threonine residue in place of N728 of PLCγ_1_, lacking positive polarity altogether. Furthermore, replacement of PLCγ_1_ N728 by alanine, effectively removing its partial positive polarity, did not relieve PLCγ_1_ from its autoinhibitory constraint [25]. The latter observation may be explained by the earlier finding that the β methylene group of PLCγ_1_ N728 shows a medium to strong NOE correlation with the α methylene group of a leucine present in position pY+4 of a tyrosine-phosphorylated peptide of the platelet derived growth factor (PDGF) receptor [29]; the latter groups would also be provided by the N728-N(pY783+4) and T706-S(pY759+4) pairs of the cSH2-intramolecular pY-peptide complexes of PLCγ_1_ and PLCγ_2_, respectively, as well as by the A728-N(pY783+4) pair of mutant PLCγ_1_.

**Figure 15:**
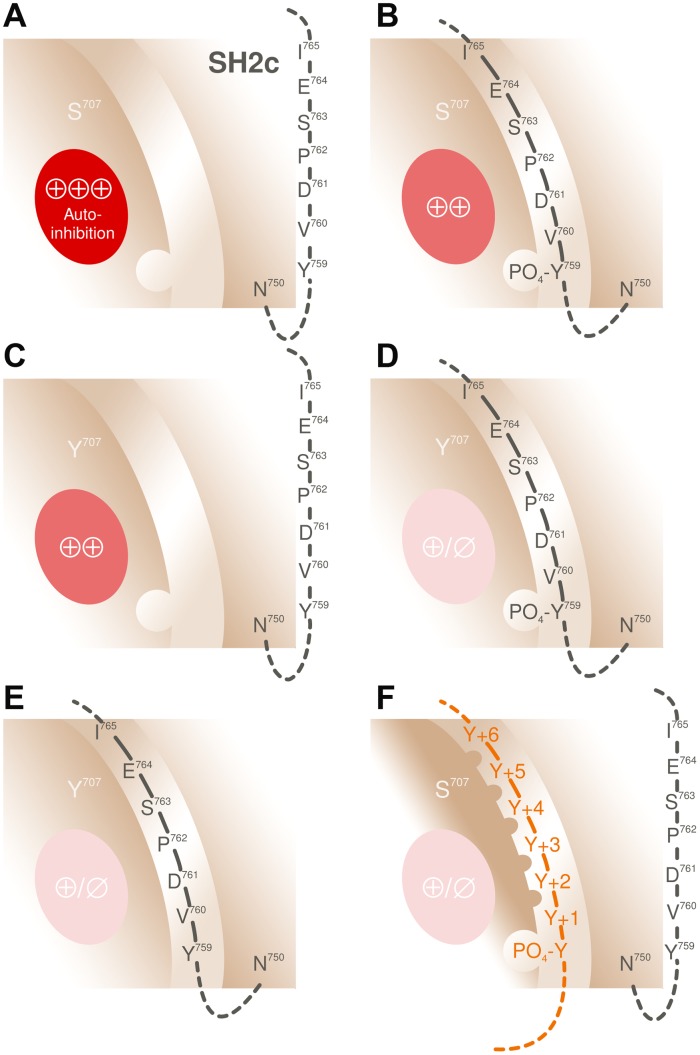
Mechanisms potentially involved in mediating activation of PLCγ_2_ through mutations in position S707 (**A**) In wild-type PLCγ_2_, the SH2c-SH3 linker peptide carrying the protein tyrosine kinase substrate Y759, does not occupy the pY-peptide-binding groove on cSH2, thus allowing for maximal autoinhibition of PLCγ_2_ activity by this domain (⊕⊕⊕). (**B**) Phosphorylation of Y759 causes an interaction of the linker peptide with its binding groove and a reduction of enzyme autoinhibition by cSH2 (⊕⊕), hence, activation of PLCγ_2_. (**C**) The activating mutations in position 707, e.g. S707Y, could cause a loss of the autoinhibitory properties of cSH2, thus obviating, at least in part, the requirement of its interaction with the pY759 peptide for enzyme activation. (**D**) Interaction of the phosphorylated linker peptide could further reduce PLCγ_2_ autoinhibition. In addition, the structural change induced by the S707 mutations could increase the affinity of the extended cSH2 groove for the pY759 peptide and thus enhance the likelihood of pY-peptide-groove-complex formation. Alternatively, the S707 mutations could alter the overall structure of the pY-peptide-groove-complex even without altering the pY-peptide-groove-interaction, such that the complex attains an even lower affinity for the electronegative counterpart on the catalytic core or a lower (residual) efficacy as an enzyme autoinhibitor (⊕/∅). Consistent with this notion, structural differences have been observed between the pY-peptide-complexed and -uncomplexed forms of the Src SH2 domain, with largest shifts seen for the EF loop residues corresponding to G705, T706, and S707 of PLCγ_2_ [48] (**E**) Since the PLCγ_1_ Y783 peptide has been shown to interact with the peptide-binding groove on PLCγ_1_ cSH2 even in the absence of Y783 phosphorylation [20], the corresponding mutations at S707 of PLCγ_2_ could enhance the binding and, hence, the functional effects of even the non-phosphorylated Y759 linker peptide. (**F**) Alteration of the pY-peptide binding specificity of PLCγ_2_. It is well known that differences between the EF loops of other SH2 domains are important determinants of the specificity of the pY-peptide-extended-groove binding. For example, Grb2-like SH2 domains contain a bulky tryptophan residue at a position corresponding to G705 of PLCγ_2_, which protrudes from the EF loop and blocks the pY+3 position, if the phosphopeptide is in an extended conformation. This forces the bound peptides to make a β-turn, which is accomplished by selecting an asparagine residue in the pY+2 position [49, 50]. Replacement of the serine residue corresponding to G705 of PLCγ_2_ in the Src SH2 domain by a tryptophan residue switched its selectivity for pY-peptides to resemble that of the Sem5/drk/Grb2 SH2 domain [51]. Pertinent to this issue, PLCγ cSH2 domains have been shown to interact with many different peptide ligands, however, they do so with a defined specificity [52–54]. Structural changes at and/or near residue S707 of PLCγ_2_ may, therefore, alter the specificity of peptide recognition and, hence, the “language” of this domain [55]. The observation that a PLCγ_2_ mutant carrying mutations expected to block activation by phosphorylation of intramolecular tyrosine residues (4F) and Rac (F897Q) was still activated by EGF is consistent with this view (*cf.* Figure 11).

How, then, could replacement of S707 by Y, F, and P, and, by extension, A708 by P, cause enhancement of basal as well as stimulated activity in intact cells? Charge alterations are unlikely to be relevant, because of the stimulatory effects of the replacements S707Y and A708P, maintaining the charge characteristics and the hydrophobicity, respectively, of the two side chains. Side chain size alterations appear more likely to be pertinent, however, leading to alterations of the EF loop surface with potential functional consequences for the interaction of the extended cSH2 groove and the Y759 peptide. With regard to the latter, several scenarios are possible, not necessarily in a mutually exclusive manner. These are shown in Figure 15, and extended upon in the corresponding legend. Identification of the relevant molecular mechanism is likely to facilitate the development of inhibitors specifically targeting S707 mutant, but not wild-type PLCγ_2_, in patients suffering from acquired tumor drug resistance or hereditary autoinflammation [30, 31].

As noted before [1, 16], the degrees of activation of the two *PLCG2* deletion mutants underlying PLAID at 37° C are relatively minor (*cf.* Figure 12). Under these conditions, the APLAID mutant S707Y shows a much more marked enhancement of basal activity. Unlike the PLAID mutants, the mutant S707Y is not activated by cooling, but instead shows a gradual decrease in inositol phosphate formation with decreasing temperature, as predicted by the Arrhenius equation [32]. PLCγ_2_S707Y also qualitatively resembles wild-type PLCγ_2_, rather than the PLAID PLCγ_2_ mutants, in terms of the temperature-dependence of the stimulatory effect of activated Rac2 (*cf.* Figure 13B and [16]).

We have previously shown that the PLAID mutants PLCγ_2_Δ19 and PLCγ_2_Δ20–22, in contrast to wild-type PLCγ_2_ are resistant to agonist activation of EGF receptors endogenously expressed in COS-7 cells [16], a finding analogous to observations made in patient and mutant transfected B-cells. In contrast, PLCγ_2_S707Y shows a markedly enhanced, further activation by EGFR in this system (*cf.* Figures 11 and 14). PLCγ_2_Δ19 and PLCγ_2_Δ20–22 lack cSH2 residues 646 to 685 and cSH2-SH3 residues 686 to 806, respectively. While the latter segment contains arginine residues R653, R672, and R674 predicted to provide the binding site on cSH2 for pY peptides, the former not only contains the two tyrosine residues phosphorylated by PLCγ-activating upstream tyrosine kinases, Y753 and Y759, but also the fourth arginine residue comprising the putative pY binding site, R694, and all constituents of the groove representing the binding site for the residues following pY in PLCγ_2_-activating pY peptides. Hence, by analogy to the “two-pronged plug two-holed socket” model for the mechanism of binding of the Src SH2 domain to pY peptides, the two PLAID deletions within PLCγ_2_ are predicted to each remove either most of one hole of the socket or the rest of this and the entire other hole as well as a major plug normally involved in *in cis* activation of the enzyme. This would clearly explain the resistance of the two mutants to EGFR stimulation. However, both the EF and the BG loop, providing the putative key elements of the autoinhibitory interaction of cSH2 with the enzyme´s catalytic core, are lacking in mutant PLCγ_2_Δ20-22, yet this mutant, similar to PLCγ_2_Δ19, shows an only marginal enhancement of basal activity at 37° C [16]. Hence, the functions of the presumed autoinhibitory cSH2 regions lacking in PLCγ_2_Δ20-22 may be taken on by other structural elements of the mutant enzyme. Alternatively, autoinhibition of the catalytic core may be more complex and rely on mechanisms in addition to cSH2-catalytic-core-interaction. In any case, the functional differences between the PLAID and the APLAID PLCγ_2_ mutants shown here provides a conceptual framework for the understanding of the functional differences observed in intact immune cells such as B cells [3, 24], and may explain activation of the NLRP3 inflammasome in peripheral blood mononuclear cells of APLAID patients [14] Interestingly, the NLRP3 inflammasome plays an important role in the pathogenesis of not only relatively rare disorders such as cryopyrin-associated periodic syndromes (CAPS), but also more common diseases such as gout, type 2 diabetes mellitus, artherosclerosis, and Alzheimer's disease (referenced in [14]. This raises the possibility that alterations in PLCγ_2_ activity may be involved in these diseases as well. In line with this suggestion, a variant of *PLCG2* (rs72824905: p.Pro522Arg) has very recently been shown to be associated with protection against the development of late-onset Alzheimer´s disease [13].

Our study leaves several intriguing questions unresolved. One is, why PLCγ_2_S707Y is overactive in intact cells, but not in the reconstituted system. The most straightforward explanation for this finding is that there is no effect of the S707Y mutation on the intrinsic properties of the PLCγ_2_ enzyme, phospholipid substrate affinity and catalytic activity. Alternatively, the presentation of the substrate to the PLCγ_2_ may be different in artificial lipid vesicles than in native plasma membranes. However, we have previously demonstrated that the lipid vesicles used herein for the reconstituted system are well suited to observe PLCγ_2_ stimulation by activated Rac2 or by removal of its autoinhibitory PHn-SH2n-SH2c-SH3-PHc tandem [33]. Hence, it appears likely that the S707Y mutation stimulates the enzyme mainly by causing hypersensitivity of the enzyme to stimulatory proteins such as activated Rac or upstream protein tyrosine kinases. As to the latter, tyrosine kinases other than Btk, e.g. Syk and Lyn, which are known to interact with and activate PLCγ_2_, are attractive candidates for this role, since PLCγ_2_S707Y mediates resistance to inhibition of Btk. Of note, the R665W mutant of PLCγ_2_ has been shown to mediate functional Btk independency through a pathway(s) involving Syk and Lyn [34].

The second question is why S707Y is not activated by cooling. We have previously shown that the ability of PLCγ_2_ mutants to respond to cold temperatures resides in the two halves of the catalytic core of the enzyme (including upstream and downstream regulatory regions but excluding the SH2n-SH2c-SH3 tandem), and requires the flexibility of the two halves relative to each other [16]. Thus, we hypothesize that the Δ19 and Δ20-22 deletions of the PLAID mutants, unlike the S707Y point mutation of APLAID and CLL ibrutinib resistance, cause a loss of the rigid orientation of the catalytic dyad normally brought about by the SH2n-SH2c-SH3 tandem.

Finally and third, why does a single mutation, S707Y, of *PLCG2* cause, on the one hand, autoinflammation when occurring in the germline, with no evidence yet for increased development of B cell malignancies in the affected APLAID patients, and, on the other hand, progression of B cell malignancies to drug-resistance when occurring somatically in CLL cells? There is precedence, in the case of STAT3, for the development of cancer, mostly large granular lymphocytic (LGL) leukemias, with somatic gain of function mutations, with much less manifestation of malignancies in patients harboring the same mutations in germline cells. The latter suffer from early-onset and severe multi-organ autoimmunity and immune dysregulation (reviewed in [35]). Perhaps even more intriguingly, nonclonal eosinophilia, atopic dermatitis, urticarial rash, and diarrhea have been reported for patients with somatic, gain-of function mutation of *STAT5b* occurring early on in hematopoiesis, while mutations arising later on are highly associated with leukemia and lymphoma [36]. Genetically defined, autosomal dominant human primary immunodeficiencies have been found to manifest in various combinations of phenotypes belonging to five broad categories: autoimmunity, autoinflammation, allergy, infection, and malignancy [37]. *PLCG2*-related immunodeficiencies are associated with the former four, there is no evidence, currently, for an association with malignancies. However, activated phosphoinositide 3-kinase δ syndrome (APDS) for example, caused by activating mutations in the gene encoding the catalytic subunit of the enzyme (PI3Kδ), has recently been shown to share infection, autoinflammation, and autoimmunity with APLAID, but to also give rise to nonneoplastic lymphoproliferation and lymphoma in 75% and 13%, respectively, of 53 patients studied in a large genetically defined international APDS cohort [38]. Both cell-extrinsic causes of malignancies, such as impaired immunosurveillance, and cell-intrinsic causes like alterations of lymphocyte development, differentiation, and (co)signaling may play a role in this context [39]. In B cells, Btk is thought to mediate the functional interaction between membrane-immunoglobulin-stimulated PI3Kδ and PLCγ_2_, resulting in a strong convergence of clinical activity of the corresponding inhibitors, ibrutinib and idelalisib, in malignancies of mature B cells [40]. Thus, close monitoring of patients with autosomal dominant primary immunodeficiencies caused by *PLCG2* mutations for B cell malignancies appears advisable.

## MATERIALS AND METHODS

### Materials

The mouse monoclonal antibody 9B11 reactive against the c-Myc epitope (EQKLISEEDL) and the rabbit polyclonal antiserum reactive against human PLCγ_2_ (sc-407) were obtained from Cell Signaling Technology and Santa Cruz Biotechnology, respectively. The rabbit polyclonal antiserum reactive against human Rac2 (sc-96) was purchased from Santa Cruz Biotechnology. The anti-β-actin antibody (clone AC-15) and the anti-Rac1 antibody (clone 23A8) were obtained from Sigma and Merck Millipore, respectively. The Rac inhibitor EHT 1864 and its inactive analog EHT 4063 were synthesized as described previously [41]. Human epidermal growth factor (EGF) (E9644) was from Sigma. ProGreen baculovirus vector DNA (A1) was purchased from AB Vector.

### Construction of vectors

The construction of complementary DNAs encoding c-Myc epitope-tagged human PLCγ_2_ (1265 amino acids, accession number NP_002652), and F897Q mutant of PLCγ_2_ was described previously [42]. The 4F mutant (Y753F, Y759F, Y1197F, Y1217F) of PLCγ_2_ was prepared as described in [43]. The cDNA of PLCγ_2_Δ19 (deletion of exon 19, aa 646–685) was constructed by *in vitro* mutagenesis using the QuikChange II XL Site-Directed Mutagenesis Kit (200521, Agilent Technologies). The deletion of exons 20–22 in PLCγ_2_ (PLCγ_2_Δ20-22, aa 686–806) was performed using the PCR overlap extension method [16]. The construction of all other vectors and of the baculoviruses was outlined in Refs. [18, 43]. Complementary DNAs encoding point mutants of PLCγ_2_ were constructed by *in vitro* mutagenesis using the QuikChange II XL Site-Directed Mutagenesis Kit. The primer sequences and PCR protocols are available from the authors upon request. A vector encoding c-Myc epitope-tagged human PLCδ_1_Δ44 was kindly supplied by J. Sondek [44].

### Cell culture and transfection

The COS-7 cell line was purchased from the American Type Culture Collection (ATCC, # CRL-1651). The cells were maintained at 37° C in a humidified atmosphere of 90% air and 10% CO_2_ in Dulbecco's modified Eagle's medium (DMEM) (catalog no. 41965–039, Gibco) supplemented with 10% (v/v) fetal calf serum (catalog no. 10270–106, Gibco), 2 mM glutamine, 100 units/ml penicillin, and 100 μg/ml streptomycin (all from PAA Laboratories). Prior to transfection, COS-7 cells were seeded into 24-well plates at a density of 0.75 × 10^5^ cells/well and grown for 24 h in 0.5 ml of medium/well. For transfection, plasmid DNA (500 ng/well) was diluted in 50 μl of jetPRIME^®^ buffer, and 1 μl of jetPRIME^®^ was added according to the manufacturer's instructions. The total amount of DNA was maintained constant by adding empty vector. Four hours after the addition of the DNA-jetPRIME^®^ complexes to the dishes, the medium was replaced by fresh medium, and the cells were incubated for a further 20 h at 37° C and 10% CO_2_.

### Radiolabeling of inositol phospholipids and analysis of inositol phosphate formation

Twenty four hours after transfection, COS-7 cells were washed once with 0.3 ml/well Dulbecco's PBS (PAA Laboratories) and then incubated for 18 h in 0.2 ml/well DMEM containing supplements as described above, supplemented with 2.5 μCi/ml *myo*-[2-^3^H]inositol (NET1156005MC, PerkinElmer Life Sciences) and 10 mM LiCl. The cells were then washed once with 0.2 ml/well of and lysed by addition of 0.2 ml/well 10 mM ice-cold formic acid. The analysis of inositol phosphate formation was performed as described previously [18]. To examine EGF-mediated PLCγ_2_ stimulation, COS-7 cells were radiolabeled for 24 h in serum-free DMEM as described previously [45]. Briefly, cells were washed twice with 0.3 ml/well DMEM containing the above supplements except serum and then incubated for 24 h in 0.2 ml/well of the same medium supplemented with 0.25% fatty-acid-free bovine serum albumin (A8806, Sigma) and 2.5 μCi/ml *myo*-[2-^3^H]inositol. The cells were then washed with 0.3 ml/well Dulbecco's PBS and incubated for 1 h in 0.2 ml/well DMEM without serum containing the above supplements, 20 mM LiCl, and increasing concentrations of EGF. To examine cold-temperature-mediated PLCγ_2_ stimulation, radiolabeled COS-7 cells were incubated for 18 h in six identical Midi 40 CO_2_ Incubators (Thermo Fisher Scientific) in humidified atmospheres of 90% air and 10% CO_2_ at temperatures ranging from 27° C to 37° C. After removal of the medium, the cells were lysed by addition of 0.2 ml/well of 10 mM ice-cold formic acid for analysis of inositol phosphate formation.

### Measurement of PLCγ_2_ activity *in vitro*

The production of soluble fractions of Sf9 cells containing c-Myc epitope tagged PLCγ_2_ and PLCγ_2_S707Y and the determination of PLC activity *in vitro* were carried out as described previously [18].

### Determination of the 10-degree temperature coefficients

According to Hille [46], the 10-degree temperature coefficient, *Q*_10_, of a biological process can be calculated for an arbitrary temperature interval Δ*T* from

$$Q_{\Delta T}={\left(Q_{10}\right)}^{\Delta T/10}$$

Using Δ*T* = *T_i_*–*T_ref_* and $Q_{\Delta T}=\frac{A_{i}}{A_{ref}}\;\;$, where *T_i_* and *T_ref_* are the individual and a reference temperatures and *A_i_* and *A_ref_* are the individual and a reference activity, this equation can be rewritten to

$$log_{10}\left(\frac{A_{i}}{A_{ref}}\right)=0.1\left(T_{i}-T_{ref}\right)log_{10}\left(Q_{10}\right)$$

$$log_{10}\left(\frac{A_{i}}{A_{ref}}\right)=0.1log_{10}\left(Q_{10}\right)T_{i}-0.1log_{10}\left(Q_{10}\right)T_{ref}$$

Upon plotting the *T*_i_ *vs.* $log_{10}\left(\frac{A_{i}}{A_{ref}}\right)$, the *Q*_10_ value(s) can be calculated from the slopes of the linear portions of the resultant graphs.

### Miscellaneous

SDS-PAGE and immunoblotting were performed according to standard protocols using antibodies reactive against the c-Myc epitope for wild-type and mutant PLCγ_2_. Immunoreactive proteins were visualized using the Pierce ECL Western blotting detection system (#32106, ThermoFisher Scientific). Samples to be analyzed by Western blotting were taken, *quasi* as a fourth replicate, from the same plate as and immediately adjacent to the samples taken in triplicate for functional analysis. Using this protocol and paying meticulous attention to experimental detail, we have not experienced variations in gel loading of these samples. All experiments were performed at least three times. Similar results and identical trends were obtained each time. Data from representative experiments are shown as means ± S.E. of triplicate determinations. In Figure 1, 2, 3B, 4, 8, 9A, 9C, 10B, 11, and 13, the data were fitted by nonlinear least squares curve fitting to three- or four parameter dose-response equations using GraphPad Prism, version 5.04. In certain cases, the global curve fitting procedure contained in Prism was used to determine whether the best fit values of selected parameters differed between data sets. The simpler model was selected unless the extra sum of squares *F*-test had a *p* value of less than 0.05.

## SUPPLEMENTARY MATERIALS FIGURES AND TABLES



## Abbreviations

APLAID: autoinflammation and PLCγ_2_–associated antibody deficiency and immune dysregulation
Btk: Bruton´s tyrosine kinase
CLL: chronic lymphocytic leukemia
ERK: extracellular-signal regulated kinase
GEF: guanine nucleotide exchange factor
GTPγS: guanosine 5′-(3-*O*-thio)triphosphate
PLAID: PLCγ_2_–associated antibody deficiency and immune dysregulation
PLC: phospholipase C
PtdIns*P*_2_: phosphatidyl-inositol 4,5-bisphosphate
Rac: Ras-related C3 botulinum toxin substrate
cSH2: C-terminal Src homology domain 2
nSH2: N-terminal Src homology domain 2
SH3: Src homology domain 3
spPH: split pleckstrin homology domain.
