# Supplementary material for: Functional characterization of phospholipase C-γ2 mutant protein causing both somatic ibrutinib resistance and a germline monogenic autoinflammatory disorder
Source: Oncotarget. 2018 Sep 28;9(76):34357–78. doi: 10.18632/oncotarget.26173 (PMC6188132; doi:10.18632/oncotarget.26173)
Supplement: Supplementary file 1 [file oncotarget-09-34357-s001.pdf]

Functional characterization of phospholipase C-γ_2 mutant protein causing both somatic ibrutinib resistance and a germline monogenic autoinflammatory disorder

SUPPLEMENTARY MATERIALS

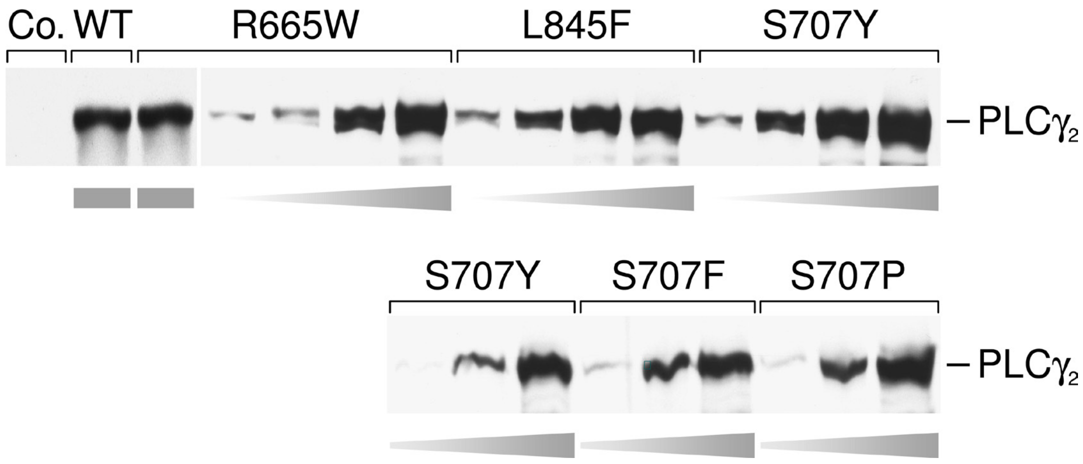

Supplementary Figure 1: Expression of wild-type and mutant PLCγ_2 isozymes in the experiment shown in Figure 1. Cells from one well each were washed with 0.2 ml of Dulbecco's PBS and then lysed by addition of 100 µl of SDS-PAGE sample preparation buffer. Aliquots of the samples were subjected to SDS-PAGE and immunoblotting was performed using an antibody reactive against the c-Myc epitope present on wild-type and mutant PLCγ_2.

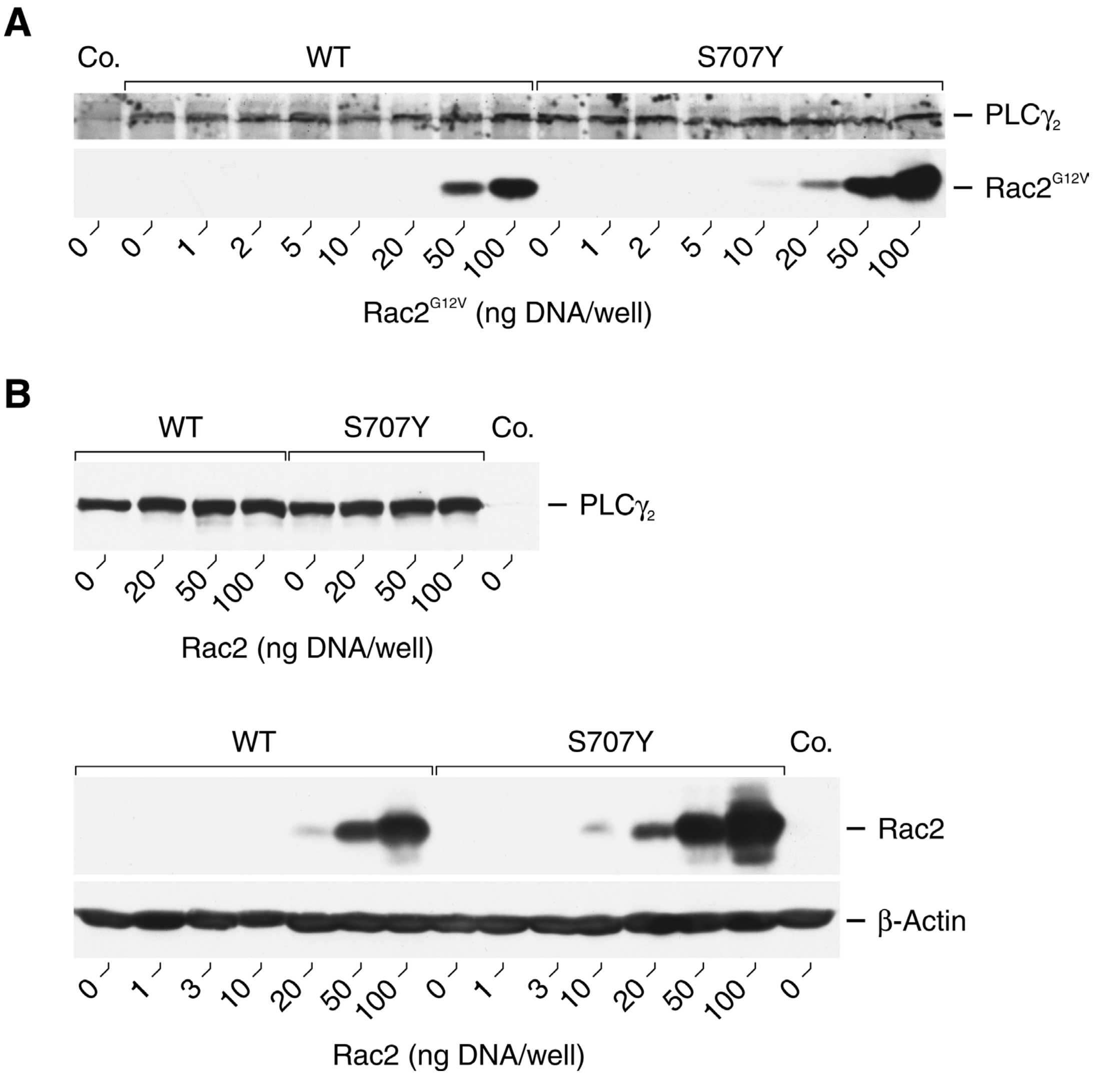

Supplementary Figure 2: Expression of wild-type and mutant PLCγ$_2$ isozymes and Rac2 in the experiment shown in Figure 2. Cells from one well each were analyzed by SDS-PAGE and immunoblotting using an antibody reactive against the c-Myc epitope, antibody reactive against Rac2 or β-actin. *Co.,* control.

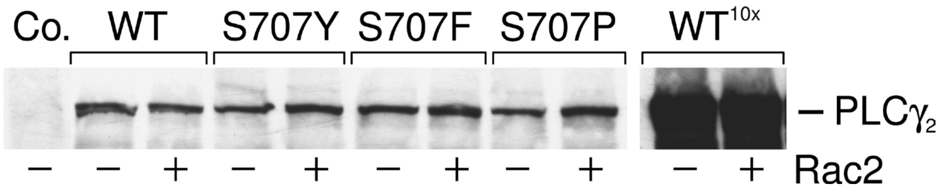

Supplementary Figure 3: Expression of wild-type and mutant PLCγ_2 isozymes in the experiment shown in Figure 3. Cells from one well each were analyzed by SDS-PAGE and immunoblotting using an antibody reactive against the c-Myc epitope.

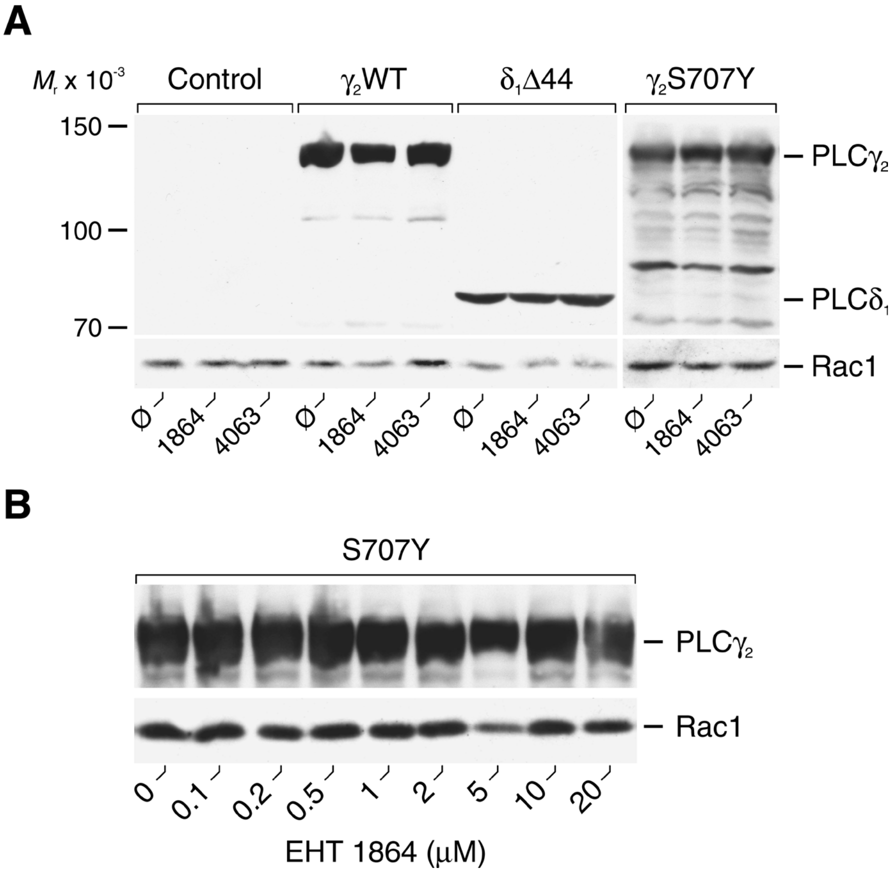

Supplementary Figure 4: Expression of exogenous wild-type and mutant PLCγ_2 isozymes, PLC$\delta_1\Delta$44 and endogenous Rac1 in the experiment shown in Figure 4. Cells from one well each were analyzed by SDS-PAGE and immunoblotting using an antibody reactive against the c-Myc epitope present on wild-type and mutant PLCγ_2 as well as on PLC$\delta_1\Delta$44 or antibody reactive against Rac1 endogenously present in COS-7 cells.

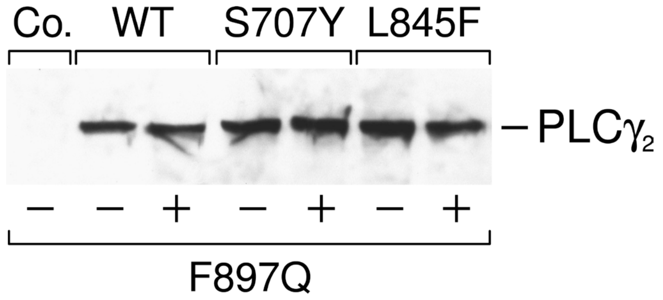

Supplementary Figure 5: Expression of wild-type and mutant PLCγ2 isozymes in the experiment shown in Figure 5. Cells from one well each were analyzed by SDS-PAGE and immunoblotting using an antibody reactive against the c-Myc epitope.

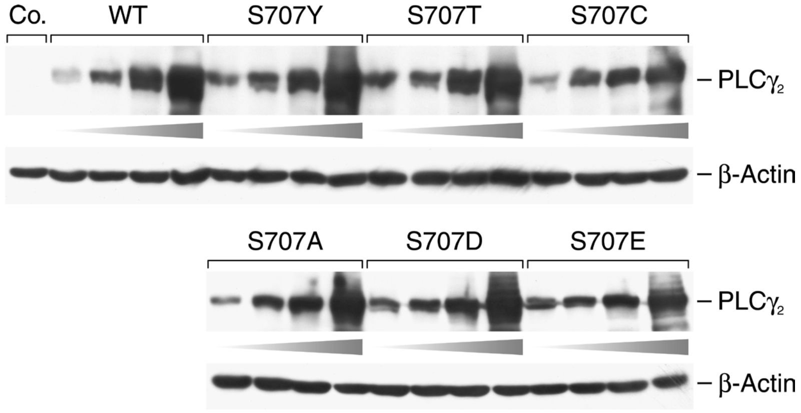

Supplementary Figure 6: Expression of wild-type and mutant PLCγ_2 isozymes in the experiment shown in Figure 7. Cells from one well each were analyzed by SDS-PAGE and immunoblotting using an antibody reactive against the c-Myc epitope present on wild-type and mutant PLCγ_2 or antibody reactive against β-actin.

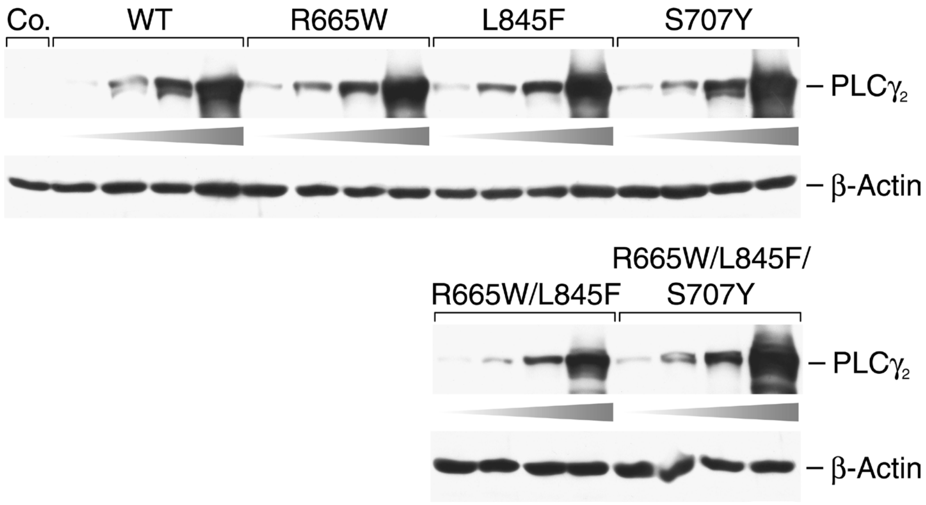

Supplementary Figure 7: Expression of wild-type and mutant PLCγ_2 isozymes in the experiment shown in Figure 8. Cells from one well each were analyzed by SDS-PAGE and immunoblotting using an antibody reactive against the c-Myc epitope present on wild-type and mutant PLCγ_2 or antibody reactive against β-actin.

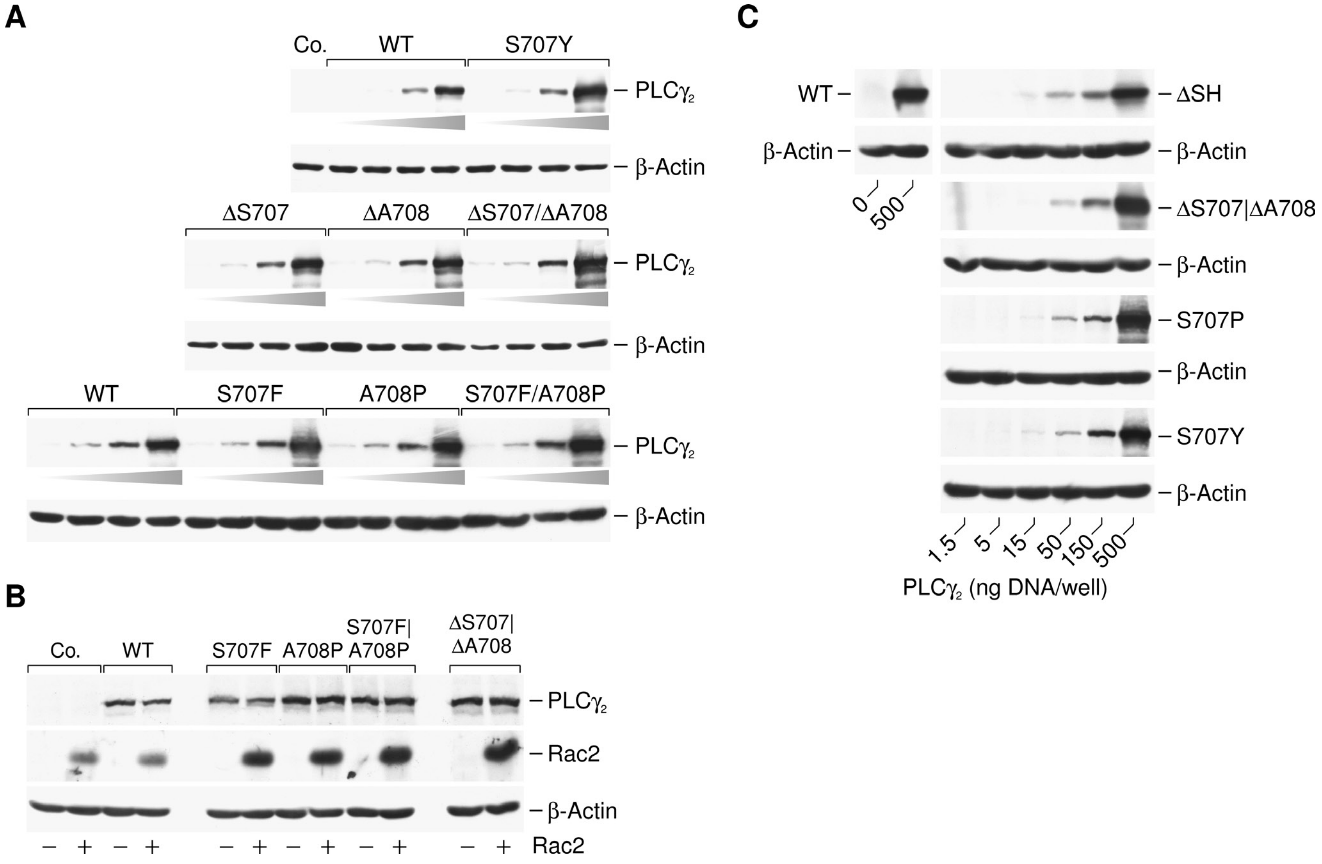

Supplementary Figure 8: Expression of wild-type and mutant PLCγ_2 isozymes in the experiment shown in Figure 9A–9C. Cells from one well each were analyzed by SDS-PAGE and immunoblotting using an antibody reactive against the c-Myc epitope present on wild-type and mutant PLCγ_2 or antibody reactive against β-actin (**A–C**) or antibody reactive against Rac2 (**B**).

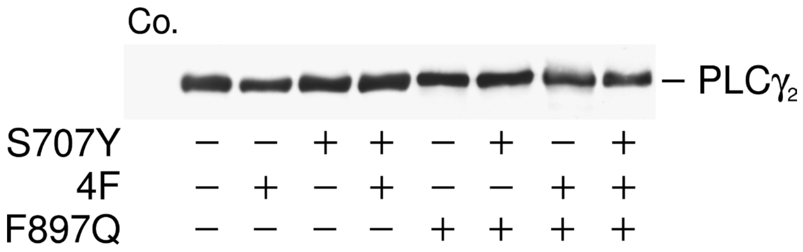

Supplementary Figure 9: Expression of wild-type and mutant PLCγ_2 isozymes in the experiment shown in Figure 11. Cells from one well each were analyzed by SDS-PAGE and immunoblotting using an antibody reactive against PLCγ_2.

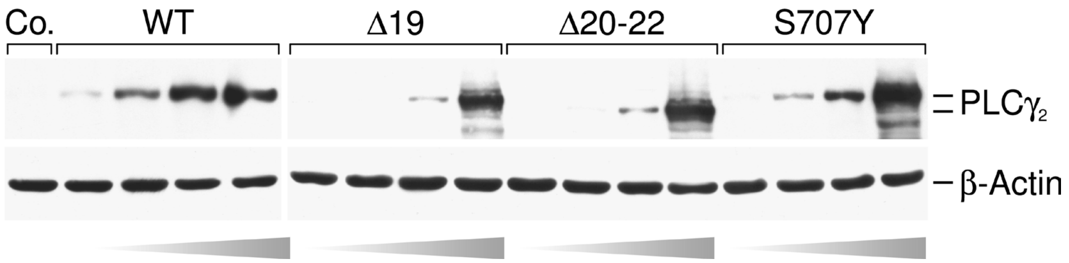

Supplementary Figure 10: Expression of wild-type and mutant PLCγ_2 isozymes in the experiment shown in Figure 12. Aliquots of the samples were subjected to SDS-PAGE and immunoblotting was performed using an antibody reactive against the c-Myc epitope present on wild-type and mutant PLCγ_2 or antibody reactive against β-actin.

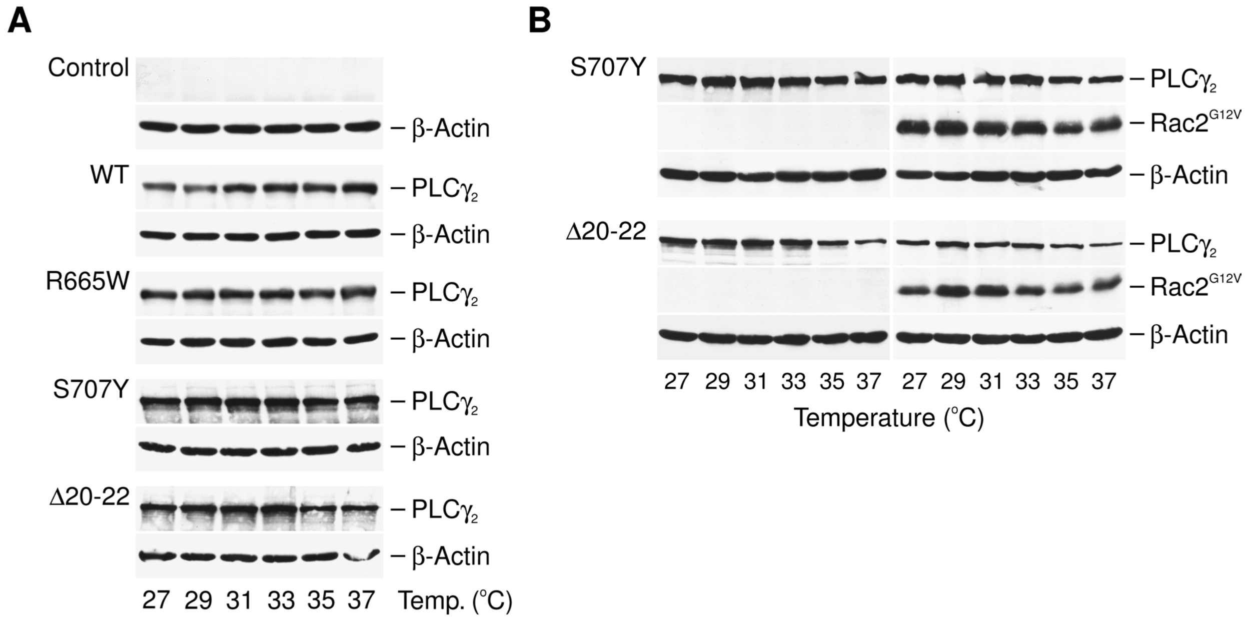

Supplementary Figure 11: Expression of wild-type and mutant PLCγ_2 isozymes in the experiment shown in Figure 13. Cells from one well each were analyzed by SDS-PAGE and immunoblotting using an antibody reactive against the c-Myc epitope present on wild-type and mutant PLCγ_2 or antibody reactive against β-actin (**A**) or antibody reactive against Rac2 (**B**).

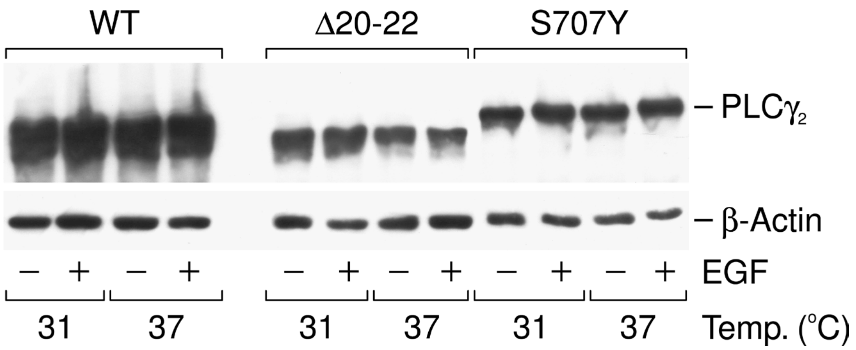

Supplementary Figure 12: Expression of wild-type and mutant PLCγ_2 isozymes in the experiment shown in Figure 14. Cells from one well each were analyzed by SDS-PAGE and immunoblotting using an antibody reactive against the c-Myc epitope present on wild-type and mutant PLCγ_2 or antibody reactive against β-actin.
